# Clinical characteristics and treatment strategies for A20 haploinsufficiency in Japan: a national epidemiological survey

**DOI:** 10.3389/fimmu.2025.1548042

**Published:** 2025-06-12

**Authors:** Mayuka Shiraki, Saori Kadowaki, Yuki Miwa, Kenichi Nishimura, Yuta Maruyama, Dai Kishida, Kazuo Imagawa, Chie Kobayashi, Hidetoshi Takada, Kanako Mitsunaga, Yuzaburo Inoue, Takasuke Ebato, Takayuki Miyamoto, Eitaro Hiejima, Shuzo Sato, Kiyoshi Migita, Tadashi Matsubayashi, Daisuke Kobayashi, Eriko Hasegawa, Utako Kaneko, Takashi Ishikawa, Masafumi Onodera, Kohei Matsushita, Yuhki Koike, Hiroaki Umebayashi, Fumihiko Kakuta, Daiki Abukawa, Yasutomo Funakoshi, Masataka Ishimura, Yusuke Otani, Takuya Nishizawa, Takashi Ishige, Reiko Hatori, Seiji Tanaka, Shouichirou Kusunoki, Kimitoshi Nakamura, Harumi Shirai, Yoshiho Hatai, Futaba Miyaoka, Shuya Kaneko, Asami Shimbo, Masaki Shimizu, Hirokazu Kanegane, Motomu Hashimoto, Nobuo Negoro, Taro Yoshida, Yasunori Wada, Masaaki Usami, Taizo Wada, Kazushi Izawa, Takahiro Yasumi, Ryuta Nishikomori, Hidenori Ohnishi

**Affiliations:** ^1^ Department of Pediatrics, Gifu University Graduate School of Medicine, Gifu, Japan; ^2^ Department of Early Diagnosis and Preventive Medicine for Rare Intractable Pediatric Diseases, Graduate School of Medicine, Gifu University, Gifu, Japan; ^3^ Department of Pediatrics, Yokohama City University Graduate School of Medicine, Yokohama, Japan; ^4^ Department of Pediatrics, Shinshu University School of Medicine, Matsumoto, Japan; ^5^ Department of Medicine (Neurology and Rheumatology), Shinshu University School of Medicine, Matsumoto, Japan; ^6^ Department of Child Health, Institute of Medicine, University of Tsukuba, Tsukuba, Japan; ^7^ Department of Allergy and Rheumatology, Chiba Children’s Hospital, Chiba, Japan; ^8^ Department of General Medical Science, Graduate School of Medicine, Chiba University, Chiba, Japan; ^9^ Department of Pediatrics, Kitasato University School of Medicine, Sagamihara, Japan; ^10^ Department of Pediatrics, Kyoto University Graduate School of Medicine, Kyoto, Japan; ^11^ Department of Rheumatology, Fukushima Medical University School of Medicine, Fukushima, Japan; ^12^ Department of Pediatrics, Seirei Hamamatsu General Hospital, Hamamatsu, Japan; ^13^ Division of Clinical Nephrology and Rheumatology, Niigata University Graduate School of Medical and Dental Sciences, Niigata, Japan; ^14^ Department of Pediatrics, Niigata University Graduate School of Medical and Dental Sciences, Niigata, Japan; ^15^ Division of Immunology, National Center for Child Health and Development, Tokyo, Japan; ^16^ Department of Gastrointestinal and Pediatric Surgery, Mie University Graduate School of Medicine, Tsu, Mie, Japan; ^17^ Department of Rheumatism, Infectious Disease, Miyagi Children’s Hospital, Sendai, Japan; ^18^ Department of Gastroenterology and Hepatology, Miyagi Children’s Hospital, Sendai, Japan; ^19^ Department of Pediatrics, Graduate School of Biomedical Sciences, Nagasaki University, Nagasaki, Japan; ^20^ Department of Pediatrics, Graduate School of Medical Sciences, Kyushu University, Fukuoka, Japan; ^21^ Department of Pediatrics, Gunma University Graduate School of Medicine, Maebashi, Japan; ^22^ Department of Pediatrics and Child Health, Kurume University School of Medicine, Kurume, Japan; ^23^ Department of Pediatrics, Faculty of Life Sciences, Kumamoto University, Kumamoto, Japan; ^24^ Department of Rheumatology and Allergology, Japanese Red Cross Medical Center, Tokyo, Japan; ^25^ Department of Pediatrics, Tokyo Bay Urayasu-Ichikawa Medical Center, Urayasu, Japan; ^26^ Department of Pediatrics, Kawaguchi Municipal Medical Center, Saitama, Japan; ^27^ Department of Pediatrics and Developmental Biology, Institute of Science Tokyo, Tokyo, Japan; ^28^ Department of Child Health and Development, Institute of Science Tokyo, Tokyo, Japan; ^29^ Department of Clinical Immunology, Graduate School of Medicine, Osaka Metropolitan University, Osaka, Japan; ^30^ Department of Pediatrics, School of Medicine, Iwate Medical University, Yahaba, Japan; ^31^ Department of Pediatrics, School of Medicine, Institute of Medical, Pharmaceutical and Health Sciences, Kanazawa University, Kanazawa, Japan; ^32^ Japan Environment and Children’s Study (JECS) Kyoto Regional Center, Kyoto University Graduate School of Medicine, Kyoto, Japan; ^33^ Clinical Genetics Center, Gifu University Hospital, Gifu, Japan; ^34^ Center for One Medicine Innovative Translational Research, Gifu University, Gifu, Japan; ^35^ Department of Laboratory of Intractable and Rare Diseases, Graduate School of Medicine, Gifu University, Gifu, Japan

**Keywords:** A20 haploinsufficiency, TNFAIP3, autoinflammatory disease, molecular target drugs, secondary failure

## Abstract

**Background:**

The severity of A20 haploinsufficiency (HA20) varies, with no established clinical guidelines for treatment. This study aimed to elucidate the clinical characteristics of, and the efficacy of treatments attempted in, patients with HA20 in Japan.

**Methods:**

Clinical information on HA20 patients from medical records was retrospectively collected through the attending physicians.

**Results:**

Seventy-two HA20 patients were identified in Japan. And, 54 patients from 37 unrelated families were analyzed in detail. HA20 patients exhibited common features, including recurrent fever, gastrointestinal and musculoskeletal symptoms, and autoimmune disease; various organ disorders (e.g. neurological, liver, and pulmonary diseases) were less common complications. Molecular target drugs (MTDs) were administered in 44.4% of patients, among which anti-tumor necrosis factor (TNF)-α agents showed efficacy in 59.5% of patients. Eleven patients did not experience control of inflammation with initial MTDs, most commonly because of relapse due to secondary failure of MTDs. Anti-drug antibodies were related to the secondary failure of adalimumab in one patient and infusion reactions to infliximab in two patients. In such refractory cases, other treatments (e.g. switching the first MTD to an alternative agent or adding a Janus kinase inhibitor or immunomodulators, or allogeneic hematopoietic cell transplantation [HCT]) were attempted.

**Conclusions:**

Our survey revealed that anti-TNF-α agents showed high efficacy. However, secondary failure of MTDs was a significant refractory-related factor in HA20 patients in Japan. Although anti-interferon therapies, thalidomide, and HCT might be potential treatment options, the results of this study suggest that further research is necessary to establish suitable treatments for HA20, especially for those with refractory disease.

## 1 Introduction

A20 haploinsufficiency (HA20) is a hereditary autoinflammatory disease caused by heterozygous loss-of-function variants in the *TNFAIP3* gene (1). The A20 protein is involved in ubiquitin modification, exhibiting suppression of two pathways: nuclear factor κ light-chain enhancer of activated B cells (NF-κB) signaling and interferon (IFN) regulatory factor signaling related to type I IFN production (2). HA20 attenuates the inhibitory effects of these signaling pathways, leading to overproduction of proinflammatory cytokines and subsequently inducing a state of systemic inflammation. Consequently, patients with HA20 present with autoinflammatory Behcet’s disease (BD)-like symptoms and may also develop autoimmune diseases (3).

The severity of HA20 varies, with no established clinical guidelines for treatment. For severe cases, administration of molecular target drugs (MTDs), such as anti-tumor necrosis factor (TNF)-α agents, anti-interleukin (IL)-1 agents, and Janus kinase (JAK) inhibitors, is reportedly effective (1, 3, 4). By contrast, MTDs were found to be ineffective or insufficiently effective in patients with refractory disease (5). Therefore, it is necessary to elucidate the clinical characteristics of, and suitable treatment methods for, HA20 patients. Herein, we conducted an epidemiological survey of the accumulated data and treatment strategies used in 54 patients with HA20 in Japan. Additionally, we focused on HA20 patients with refractory disease and analyzed their characteristics and treatments.

## 2 Methods

### 2.1 Patients and clinical information

Records of diagnosed or suspected cases of HA20 were extracted from the registry of the Primary Immunodeficiency Database in Japan (PIDJ) (6), comprising patients who were treated at hospitals collaborating with the PIDJ project or were referred for consultation to the Japanese Society for Immunodeficiency and Autoinflammatory Disease. The diagnosis of HA20 was confirmed via functional analysis of the *TNFAIP3* variant. Questionnaires were sent to the attending physicians for each case, and the following clinical information was retrospectively obtained from medical records: demographics, clinical symptoms, laboratory data, initial diagnosis, treatment, and treatment effects. Treatment effects were evaluated by the attending physician as “effective” (defined as a condition in which symptoms improved to the extent that additional treatment was not required), “improvement”, or “ineffective”. In this study, “refractory patients” were defined as those who required a change of the initial MTD to alternative agents. Details on the other methods (e.g. genetic analysis, *in vitro* functional evaluation of *TNFAIP3* variants, minigene splicing assay, evaluation of type I IFN scores, and assays of anti-drug antibodies) are described in the **Supplementary Material**.

### 2.2 Ethics approval

The ethics committees of Kyoto University and Gifu University approved this national study (protocol numbers R2259 and 29–322 and 2023-005, respectively), which was conducted in accordance with the principles of the Declaration of Helsinki. Written informed consent was obtained from individual adult patients or the legal guardian/next of kin of minor patients. Where it was difficult to contact individual adult patients or the legal guardian/next of kin of minor patients directly, or in patients who consented to the secondary use of their clinical information (e.g. during genetic testing), informed consent was obtained through an opt-out process.

## 3 Results

### 3.1 Patients

We obtained questionnaire responses from 54 patients (37 families) with HA20 who consented to our study (**Table 1**). The pedigrees of these 37 families are shown in **Supplementary Figure 1**. Records of 37 families with HA20 were examined to estimate the existence of a total of 72 patients with pathogenic *TNFAIP3* variants in Japan. Of these, 70 patients had or were theorized to have germline heterozygous variants while two had somatic mosaicism. The excluded cases and low frequency somatic *TNFAIP3* mosaicism are described in the **Supplementary Material**.

**Table 1:** Demographics of 54 patients with A20 haploinsufficiency in Japan.

Demographics	Patients, n
Number of patients	54
Number of families	37
Sex	
male	25
female	29
Age at onset	
0–5 years	35
6–10 years	9
11–15 years	3
16–20 years	4
>21 years	1
unknown	2
Patient origin	
Japanese	53
Vietnamese	1

Fifty-three of the patients were Japanese and one was Vietnamese (patient 51). Twenty-five of 54 patients were male and 29 were female, with no sex-specific difference observed. The median age of onset was 2.8 years (range: 0–27 years), with approximately 60% of patients showing onset before age 5 years (**Table 1**, **Supplementary Table 1**).

### 3.2 *TNFAIP3* variants and their pathogenicity

**Figure 1:**
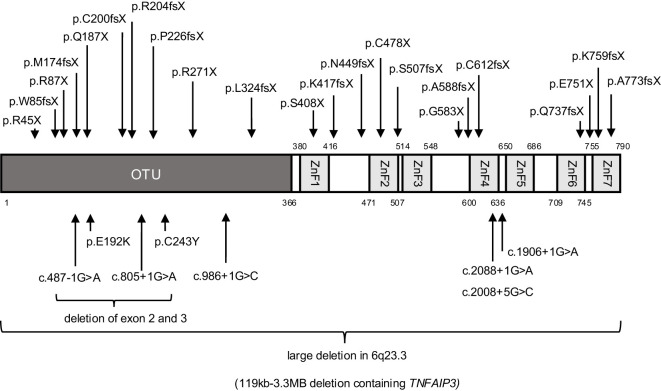
Domain structure of TNFAIP3 (A20) and sites of variation identified in 54 patients with A20 haploinsufficiency in Japan. OTU, operational taxonomic unit; ZnF, zinc finger domain.

**Figure 2:**
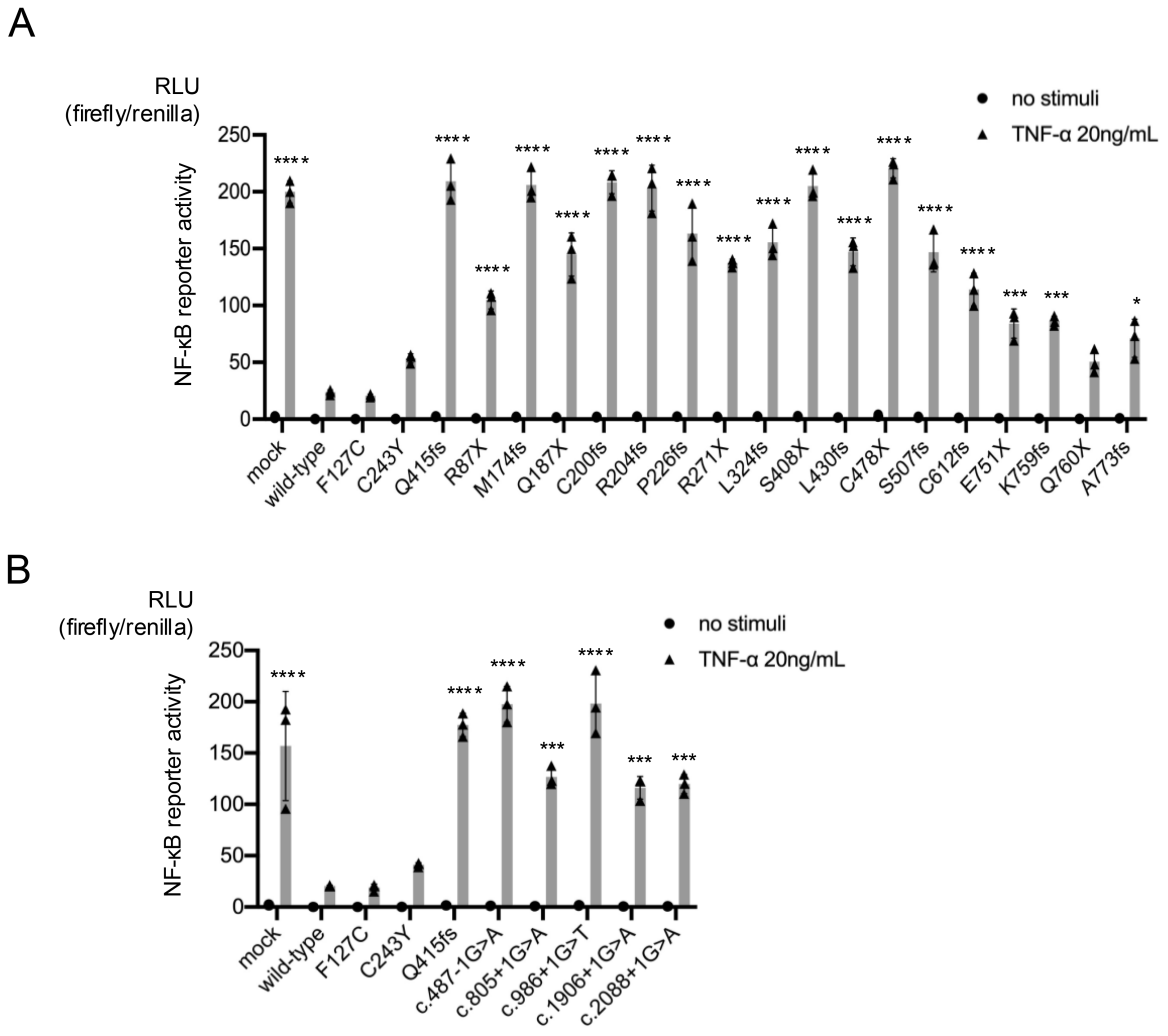
Reporter gene activity of truncating *TNFAIP3* variants. The inhibitory effects of the wild-type gene and each variant on nuclear factor (NF)-κB reporter gene activity were compared. The pathogenicity of the truncating variants was examined using an NF-κB-driven luciferase reporter gene activity assay in A20-deficient HEK293 cells stimulated with tumor necrosis factor (TNF)-α (20 ng/mL). The wild-type gene had an inhibitory effect on NF-κB reporter gene activity, while the pathological variants had a disrupted inhibitory effect. Activities of frameshift and nonsense variants **(A)** and splice variants **(B)** of *TNFAIP3* (A20). *P < 0.05, ***P < 0.001, ****P < 0.0001, as determined by one-way analysis of variance with Tukey’s multiple comparison test. RLU, relative light unit.

All 54 patients analyzed had heterozygous variants in *TNFAIP3*, comprising a total of 35 distinct pathogenic variants. The domain structure of A20 and the sites of *TNFAIP3* variation in our survey are shown in **Figure 1**. Additionally, genetic analysis of family members revealed two cases of low frequency somatic mosaicism of *TNFAIP3* in peripheral blood, with 10.06% in the mother of patient 3 and 16.7% in the father of patient 18 (3). The results of functional analyses of newly identified *TNFAIP3* variants are shown in **Figure 2** and **Supplementary Figure 2**.

### 3.3 Clinical characteristics

The 54 patients investigated all were symptomatic. Additionally, family analysis revealed that three cases with heterozygous variants were asymptomatic (penetrance: 95.7%). The two patients with somatic mosaicism of *TNFAIP3* variants were asymptomatic. **Table 2** and **Supplementary Table 2** show the clinical characteristics of the 54 HA20 patients that were surveyed. Recurrent fever, recurrent stomatitis, and gastrointestinal symptoms were common features, followed by cutaneous symptoms, musculoskeletal symptoms, genital ulcers and autoimmune diseases. Central nervous system (CNS) involvement was observed in 5 patients. Patients 10 and 33 presented with aseptic meningitis, with patient 10 showing elevated IL-6 in the cerebrospinal fluid. Patient 38 developed acute encephalopathy with seizures and impaired consciousness. Patient 44 developed dysarthria and severe seizures, with magnetic resonance imaging (MRI) of the head showing lacunar infarction with leukoencephalopathy. Patient 47 had diplopia and hiccups with cerebral white matter lesions on head MRI with elevated IL-6 in the cerebrospinal fluid. Other symptoms thought to be associated with HA20 included eight patients with lymphadenopathy/lymphadenitis, eight with liver disease (including three patients with autoimmune hepatitis [AIH] or suspected AIH), one with Hodgkin’s lymphoma and craniopharyngioma (patient 8). Pulmonary disease was observed in three patients, in whom chest computed tomography examinations revealed unidentified pneumonia with nodular and granular shadow (patient 10), granular shadows with consolidation and bronchiectasis (patient 47), and multiple small nodules (patient 54), respectively.

**Table 2:** Clinical features of 54 patients with A20 haploinsufficiency in Japan.

Feature	Patients, n (%)
Recurrent fever	46 (85.2)
Recurrent stomatitis	42 (77.8)
Gastrointestinal symptoms	41 (75.9)
Cutaneous symptoms	27 (50.0)
Musculoskeletal symptoms	20 (37.0)
Genital ulcer	19 (35.2)
Autoimmune disease	15 (27.8)
Neurological symptoms	10 (18.5)
Ocular symptoms	6 (11.1)
Cardiovascular lesions	2 (3.7)
Autoimmune disease (n)	
Hashimoto’s disease (8), Graves’ disease (1), SLE (3), SS (2), AIH* (3), AIHA and ITP (1), psoriatic arthritis (1), type 1 diabetes mellitus (1)	
Central nerve system involvements and Neurological symptoms (n)	
aseptic meningitis (2), acute encephalopathy (3), lacunar infarction (1), seizures (2), dysarthria and paralysis (1) diplopia and hiccups (1), headache (5), core ataxia (1), psychiatric symptoms (1)	
Ocular disease and symptoms (n)	
uveitis (1), posterior iris adhesions apparently resulting from uveitis (1), conjunctival congestion (1), eyelid conjunctival aphthae (1), floaters (1), dry eyes (1), impaired vision and diplopia (1)	
Cardiovascular lesions (n)	
aortic regurgitation (1), myocarditis (1)	
Symptoms thought to be associated with HA20 (n)	
lymphadenopathy/lymphadenitis (8), liver disease (8), renal urological disease (4), pulmonary disease (3), persistent EBV DNA in peripheral blood (1), Hodgkin’s lymphoma and craniopharyngioma (1), BCG dermatitis and cheilitis (1), sensorineural hearing loss (1)	
Medical history	
PFAPA* (1), IgA vasculitis (2), nephrotic syndrome (1), hemophagocytic lymphohistiocytosis (1)	

*Including suspected patients. AIH, autoimmune hepatitis; AIHA, autoimmune hemolytic anemia; ALPS, autoimmune lymphoproliferative syndrome; BCG, bacille Calmette-Guerin; EBV, Epstein-Barr Virus; IgA, immunoglobulin A; ITP, immune thrombocytopenia; PFAPA, periodic fever, aphthous stomatitis, pharyngitis, and adenitis; SLE, systemic lupus erythematosus; SS, Sjogren’s syndrome.

Interestingly, patients carrying the same pathogenic variant exhibited different phenotypes and varying degrees of severity. Regarding phenotypes, in family 27, which carried the p.A773fs variant, the younger brother (patient 43) experienced recurrent episodes of fever and lymphadenopathy from 3 months of age, whereas the elder brother (patient 44) developed recurrent fevers and severe neurological symptoms in adulthood. Regarding severity, in three families (families 1, 3 and 7), patients 1, 4 and 16 presented with refractory disease, whereas other members with pathogenic variants exhibited only mild symptoms. Additionally, the severity differed between patient 3 (c.2088 + 5G>C) and patient 49 (c.2099 + 1G>A), despite both exhibiting exon 8 skipping (p.H636fsX).

### 3.4 Laboratory data

Inflammation markers were elevated in many patients during flares, with an average white blood cell count of 11,886/μl (range 600-23,380) and an average C-reactive protein (CRP) level of 7.89 mg/dl (range 0.31-19.89). Additionally, during non-flare periods, the average CRP level was 0.44 mg/dl (range 0-6.42), with nine patients testing positive. Liver enzymes were elevated in eight patients, and patient 53 had aspartate aminotransferase (AST) and alanine aminotransferase (ALT) levels of 1,659 and 1,446 U/L, respectively.

Human leukocyte antigen (HLA) data were examined in 31 patients, with HLA-A26 confirmed in five patients, B27 in one patient, B51 in six patients, and B52 in four patients. Patient 3, who required hematopoietic cell transplantation (HCT), was both HLA-B51-positive and HLA-B27-positive. Nineteen patients were evaluated for the type I IFN score, of whom 16 patients had an elevated IFN score and three patients carrying missense variants p.E192K or p.C243Y exhibited IFN levels within the normal range (**Supplementary Table 2**, **Supplementary Figure 3**). Anti-drug antibodies were measured in 11 patients (**Supplementary Table 3**). Of these, anti-adalimumab (ADA) antibodies were positive in one patient with secondary failure of ADA and in two patients still using ADA. Anti-infliximab (IFX) antibodies were positive in two patients. Four out of five patients with positive anti-drug antibodies were treated with concomitant immunosuppressants.

Gastrointestinal endoscopy was performed in 35 patients (including capsule or small bowel endoscopy in six patients), with confirmed lesions throughout the gastrointestinal tract (**Table 3**). Most of the lesions were skip lesions, although three patients had diffuse lesions and two of them showed loss of vascular patterning or granular mucosa, as observed in ulcerative colitis. Pathological findings showed that the infiltrating inflammatory cells varied from neutrophil-dominant cases to lymphocyte- and plasma cell-dominant cases, with granulation tissue observed in four patients.

**Table 3:** Endoscopic features of 35 patients with A20 haploinsufficiency in Japan.

Disease location	Macroscopic findings	Patients (n)
Colon	Ulcers	11/32
	Erosions	10/32
	Loss of vascular pattern	2/32
	Granular mucosa	1/32
Ileocecal region	Ulcers	7/32
	Erosions	0/32
Terminal ileum	Ulcers	4/32
	Erosions	2/32
Small intestine (excluding terminal ileum)	Ulcers	2/6
	Erosions	2/6
Duodenum	Ulcers	2/15
	Erosions	0/15
Gastroesophageal	Ulcers	2/15
	Erosions	3/15

Pathological findings	Patients (n)
inflammatory cells infiltrating	
neutrophil	5/23
lymphocyte	7/23
eosinophil	3/23
Granulomas	4/23

Liver biopsy was performed in three patients with liver dysfunction. Pathology in two of these patients showed interface hepatitis and plasma cell infiltration (patients 6 and 18), consistent with AIH. Pathological findings in the third patient (patient 53) showed extensive hepatocyte loss (suspicious of atrophy of the liver parenchyma).

### 3.5 Treatments

**Figure 3:**
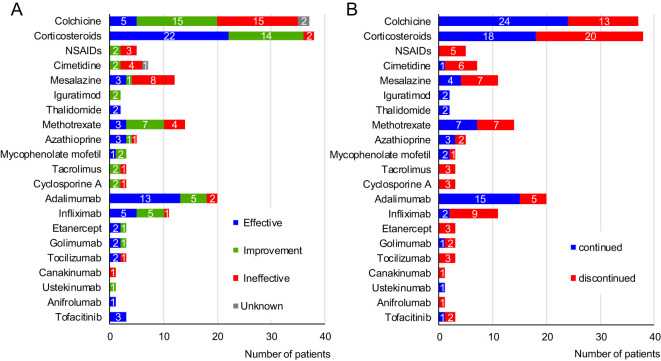
Treatments used for 54 patients with A20 haploinsufficiency in Japan. **(A)** Effects of each drug. **(B)** Continuity rate of each drug. NSAIDs, non-steroidal anti-inflammatory drugs.

The previous and current anti-inflammatory and surgical treatments and their effects in the surveyed patients are shown in **Supplementary Table 1** and **Figure 3A**. While nine of the 54 patients (16.7%) were followed without treatment, the other 45 patients (83.3%) were administered anti-inflammatory treatment. Colchicine was used in 37 patients (68.5%), as combination therapy in some, with this treatment evaluated as “effective” or “improvement” by the attending physicians of 20 patients (54.1%). Systemic corticosteroids were administered in 38 patients (70.4%), either continuously or episodically, whenever symptoms appeared, with many showing efficacy (57.9%). Thalidomide was used in combination with an MTD and was effective in two patients (patients 16 and 29). Immunosuppressants were administered to 19 patients. These immunosuppressants are still being used in 12 patients, concomitantly with MTDs in nine patients, for whom immunosuppressant monotherapy did not completely suppress the HA20 symptoms.

MTDs were administered to 24 patients (44.4%), with biologics tending to be the first choice. Among these, the anti-TNF-α agents were the most common; overall, these agents showed efficacy, with a complete response rate of 59.5%. The median duration of effective anti-TNF-α agent was 1.63 years (range 0.17-9.83). Tocilizumab was administered to three patients and was initially effective in two but was discontinued because of relapse. Canakinumab (CAN) was administered to one patient but was ineffective. Ustekinumab improved the symptoms of one patient but did not lead to remission. Anifrolumab (ANI) was effective in one patient. Tofacitinib (TOF) was administered in patients with refractory disease (see below), showing partial effectiveness (5).

Seven patients received surgical treatment: enterectomy in three patients, tonsillectomy in three patients, and craniopharyngioma removal in one patient. Enterectomy was performed for perforation of the gastrointestinal tract (patient 29), in addition to endoscopic balloon dilation for stenotic sites (patient 16). Tonsillectomy was performed in patient 20 with an initial diagnosis of periodic fever, aphthous stomatitis, pharyngitis and adenitis (PFAPA), in patient 11, in whom PFAPA symptoms persisted despite ADA therapy, and in patient 50 with recurrent histiocytic necrotizing lymphadenitis. After tonsillectomy, PFAPA symptoms disappeared in the former two cases, whereas lymphadenitis remained in the latter case.

### 3.6 The reasons for discontinuation of MTDs

Several MTD treatments were discontinued due to primary or secondary treatment failure, relapse associated with prednisolone (PSL) tapering, or adverse effects of MTDs (**Figure 3B**, **Table 4**). The most common reason for discontinuation was secondary failure of MTDs, observed in 10 agents across seven patients. The rate of concomitant immunosuppressant use did not differ between MTDs with or without secondary failure (**Supplementary Table 4**). The period from initiation of treatment to confirmation of secondary failure varied, ranging from 2 months to several years. As indicated above, one patient was positive for anti-ADA antibodies at the time of secondary failure. Additionally, IFX was discontinued because of side effects in four patients; infusion reaction in three patients, and drug-induced lupus in one patient. Three patients who had infusion reactions initially responded well to IFX but experienced a decrease in the effectiveness of IFX during the course of treatment. Anti-IFX antibodies were detectable at the onset of infusion reaction in two patients.

**Table 4:** Reasons for discontinuation of molecular target drugs.

Agents	Reasons for discontinuation of molecular target drugs (n)
Adalimumab	primary failure (2), secondary failure (4), symptoms stabilized after addition of JAK inhibitor (1)
Infliximab	primary failure (1), secondary failure (4), side effects – infusion reaction (3), drug-induced lupus (1), relapse associated with PSL reduction (1)
Etanercept	secondary failure (1), insufficient effect (1), relapse associated with PSL reduction (1)
Golimumab	insufficient effect (1), symptoms stabilized after HCT (1)
Tocilizumab	primary failure (1), secondary failure (1), relapse associated with PSL reduction (1)
Canakinumab	primary failure (1)
Anifrolumab	clinical trial discontinued due to relocation (1)
Tofacitinib	relapse associated with PSL reduction (1), side effect – generalized shingles (1)

HCT, hematopoietic cell transplantation; JAK, Janus kinase; PSL, prednisolone.

### 3.7 Treatment methods in refractory patients

The 11 patients (20.4%) with refractory disease required treatment modification to control the inflammatory conditions. The first strategy in all 11 patients was to switch the current MTD to an alternative one, which was successful in six patients. If the alternative biologic also failed to work, other agents were added: TOF in three patients, thalidomide in one patient, and an immunosuppressant in one patient. TOF was initially effective but was discontinued in 2/3 patients because of a side effect of generalized shingles and relapse associated with PSL reduction, respectively. Thalidomide was successfully introduced. One patient (patient 3) who underwent HCT because of resistance to various immunosuppressive therapies has remained in remission without anti-inflammatory therapy for 5 years post HCT.

## 4 Discussion

In this study, we described the clinical characteristics of, and the efficacy of treatments attempted in, HA20 patients in Japan. After familial Mediterranean fever and cryopyrin-associated periodic syndrome, HA20 is the third most frequent hereditary autoinflammatory disorder in Japan. Furthermore, we found that secondary failure of MTDs was a major factor contributing to refractory disease and limiting treatment options.

Regarding the characteristic findings of organ disorders, inflammatory bowel symptoms are recorded at high frequencies in HA20 patients. Endoscopically, in very-early-onset IBD, Crohn’s disease-like symptoms and colonic involvement are reportedly more common in patients with monogenic IBD than in those with non-monogenic IBD. Patients with *TNFAIP3* variants tend to show involvement of both the upper and lower gastrointestinal tract, with about 80% of patients having colonic ulcers (7). Our study confirmed these findings and also identified lesions in the small intestine. Although it is not easy to perform endoscopy in younger children, this study demonstrates the importance of confirming intestinal involvement with endoscopy in patients with gastrointestinal symptoms.

Although HA20 was originally described as a disease that presents with BD-like symptoms, subsequent reports and our investigation found that HA20 patients presented with a wide variety of symptoms indicating disorders in important organs, including neurological, liver, and pulmonary diseases and malignant lymphomas at low frequencies. Infrequent organ disorders should be noted at diagnosis and follow-up and evaluated whenever new symptoms occur. CNS involvement of HA20 includes aseptic meningitis, encephalitis/encephalopathy, CNS vasculitis, cerebral vasculopathy, and intracerebral calcifications (4, 8, 9). In this survey, elevated levels of IL-6 in the cerebrospinal fluid, which were reported as a biomarker of neuro-BD (10), were observed in two HA20 patients presenting with aseptic meningitis or encephalopathy. JAK inhibitor treatment was reportedly effective for the intracranial mass lesions in which the presence of necrotizing granulomatous inflammation was confirmed by brain biopsy (8). In our survey, three HA20 patients with encephalopathy who were started on mycophenolate mofetil or anti-TNF-α agents after being treated with corticosteroids for CNS involvement have shown no relapse of neurological disease to date. Generally, antibody products administered through normal routes are not expected to reach the CNS; thus, it is not known whether biologics are effective in the prevention or treatment of acute phase CNS involvement. Spontaneous neuroinflammation was observed in the brains of *A20* knockout and *A20* heterozygous deficient mice (11). However, spontaneous cytokine production was not the predominant cause of neurological lesions in *A20* heterozygous deficient mice, indicating that CNS lesions of HA20 patients may not be caused solely by inflammatory cytokines produced in the brain. Furthermore, in our patient, HCT improved symptoms in the skin, joints, lungs, and gastrointestinal lesions and normalized his IFN score (5), suggesting that peripheral immune cells or inflammatory cytokines produced by peripheral immune cells may influence inflammation in these organs. Studying the immune cells that directly affect neurological lesions may help to establish treatments for these lesions. A20 is a crucial hepatoprotective factor, and liver involvement (e.g. AIH) is an important form of organ damage in HA20 (12–16). The present study and previous reports suggested that acute onset or exacerbation of HA20-associated liver involvement/AIH may be ameliorated by appropriate treatment (14, 15). However, deleterious variants in *TNFAIP3* have been reported to predispose patients to AIH with cirrhosis (17), with one report of death from progressive liver disease with cirrhosis (16), necessitating careful follow-up when liver dysfunction is observed. Reports of pulmonary disease associated with HA20 have included interstitial pneumonia, nodular lesions, alveolar hemorrhage, and bronchiectasis (9, 16, 18, 19). Many cases had no respiratory symptoms associated with lung lesions, while others exhibited poor respiratory status requiring administration of MTDs (16). Given the pathogenesis of interferonopathy and vasculitis, the possibility of pulmonary involvement should be considered in HA20. Three cases of malignant lymphoma have been reported in HA20 patients (including suspected cases) in Japan: two cases with HA20 (patient 8 and the father of patient 38 (20)) and a case with suspected HA20 (the grandmother of patient 15 (3)). It has been reported that HA20 patients exhibit the skewed immune repertoire typically found in B- and T-cell lymphomas and that the risk of lymphoma may be increased in HA20 patients (21). The median age of the patients at the time of this study was 16 years; considering the age of onset of each malignant lymphoma (21, 35, and 70 years, respectively), malignant lymphoma may be an important complication in adulthood that requires attention.

The phenotype and severity of HA20 in our survey differed, even among cases with the same variant of *TNFAIP3*. HLA alleles affecting T cells are reportedly a factor influencing the phenotypes of Behcet’s spectrum disorders (e.g. recurrent aphthous stomatitis, PFAPA, and BD), including severity and tissue involvement, with the strongest association between HLA and BD (22). Although, we were unable to identify any significant associations between HLA types and the severity of HA20 (**Supplementary Table 5**), further detailed study may uncover new HLA alleles involved in HA20 phenotypes. Additionally, Karri et al. (23). noted a difference in pathogenicity between missense variants and frameshift or truncating variants of *TNFAIP3*. In this survey, there were no refractory cases among the patients with missense variants, and the type I IFN scores were lower in patients with missense variants than in those with truncating variants (**Supplementary Figure 3**), suggesting that missense variants may lead to less severe disease.

Approximately 20% of the HA20 patients surveyed had refractory disease, mainly due to secondary failure of MTD treatment. One cause of such secondary failures is thought to be the production of anti-drug antibodies, as reported in inflammatory bowel disease (IBD) and rheumatic disease (24, 25). Indeed, anti-ADA antibodies were detected at the time of secondary failure in one patient in this survey. Intriguingly, the anti-ADA antibodies were detected very early (2 months after the start of drug administration) and disappeared after ADA discontinuation (**Supplementary Table 3**). However, anti-drug antibodies were not detected at the onset of secondary failure in many of the surveyed patients, suggesting that other factors were involved in the secondary failure. Factors that reportedly influence the blood levels of IFX include polymorphism in *FCGR3A*, which is involved in the removal and excretion of human IgG1 (26), and in *FcRn*, the neonatal Fc receptor responsible for prolonging the half-life of IgG (27). Alternatively, if the blood trough level of an MTD is high enough, as it was in patient 3, the influence of inflammatory cytokines from different pathways or the presence of MTD-resistant lesions may need to be considered. Because HA20 patients experience an early disease onset and many severe patients require long-term use of MTDs, the identification of biomarkers associated with secondary failure of MTDs in HA20 is key.

For treatment, approximately 40% of HA20 patients required MTDs. Anti-TNF-α agents were the recommended first-line MTDs and the most common choice, consistent with previous reports (23, 28, 29), showing efficacy in 59.5% of patients. However, because patients may develop infusion reactions, caution should be exercised in patients with temporarily decreased efficacy during IFX administration. Regarding anti-IL-1 agents, CAN was used in only one patient and was administered during an exacerbation phase, making it difficult to evaluate the efficacy of CAN in our study. ANI was used in one patient initially diagnosed with systemic lupus erythematosus (SLE); in addition to the SLE symptoms, recurrent fever and cutaneous symptoms thought to be associated with HA20 disappeared, suggesting that ANI may be effective in the inflammatory stage of HA20 rather than JAK inhibitors. In fact, ANI efficacy was also reported in a case of the other monogenic interferonopathy, recently (30). The observation that many of our HA20 patients had increased levels of type I IFN supports a rationale for ANI as a potential therapeutic option. Moreover, the prevalence of SLE in HA20 has been reported to be 8.4% (31). ANI may be considered for HA20 patients with SLE, in whom type I IFN plays a pathogenic role.

As a treatment strategy for refractory disease, when an MTD results in primary or secondary failure, or intolerance develops, switching to an alternative MTD is recommended. Switching between anti-TNF-α agents is useful in HA20 patients, as reported in IBD (32, 33). If a biologic produced a partial effect, the addition of an immunosuppressant, thalidomide, and a JAK inhibitor were also considered as treatment options. A20 is involved in various cytokine production pathways, and which cytokines have a major influence on the pathogenesis of HA20 may vary over time and by patient. Therefore, when treating refractory disease, the response to each therapeutic agent may need to be evaluated individually and different treatment options considered. JAK inhibitors are reportedly effective in HA20 patients (8, 34). In our survey, two of three patients who received TOF discontinued TOF because of flare-ups or side effects. Possible reasons for the failure of TOF in this case include the disease being too severe during TOF administration, and the TOF-mediated suppression of type I IFN being insufficient to reduce HA20 inflammation. Therefore, the timing of its use with regard to severity of disease may need to be considered. Additionally, JAK inhibitors carry a risk of viral infection (35) and should be considered with caution. Thalidomide, which promotes TNF-α mRNA degradation and suppresses TNF-α production by monocytes, is reportedly effective in patients with intestinal BD and pediatric IBD (36–38), and in children with HA20 (39, 40). Thus, thalidomide has therapeutic value for HA20 patients, with the notable side effect of peripheral neuropathy (37, 38). HCT for HA20 has been reported in four patients, including the patient in our survey, and was effective in two patients (4, 5, 41). However, given the presence of residual disease in the two cases of successful transplantation, further study on the timing of HCT is needed. HCT has also been attempted in other autoinflammatory diseases (42), but the intensity of pretreatment and the choice of transplant source remain to be investigated.

This retrospective study had several limitations. First, anti-inflammatory therapies were often initiated in combination, especially in severe cases, making it difficult to assess the efficacy of each drug separately. Second, the timing at which the IFN score was measured varied from patient to patient. The type I IFN signature reportedly correlates with disease activity (43, 44), thus, IFN scoring at consistent time points might be helpful to determine whether type I IFN levels can be used to assess the severity of HA20. Third, early use of anti-IL-1 agents and anti-IFN agents, including JAK inhibitors and ANI, has been difficult, especially in children, in Japan. Increasing the choice of available MTDs may allow early suppression of the inflammatory stage of HA20.

In conclusion, this large-scale survey of 54 patients with HA20 in Japan revealed the involvement of multiple organs and the high efficacy of anti-TNF-α agents. However, secondary failure of MTDs was an important factor leading to refractory disease. Although anti-IFN therapies, thalidomide, and HCT might be potential treatment options, the results of this study suggest that further research is necessary to elucidate the mechanism of secondary failures and establish effective treatments for HA20, especially in patients with refractory disease.

## Data Availability

The datasets presented in this study can be found in online repositories. The names of the repository/repositories and accession number(s) can be found in the article/**Supplementary Material**.

## Collaborators

PIDJ (Primary Immunodeficiency Database in Japan) members in the JSIAD (Japanese Society for immunodeficiency and Autoinflammatory Diseases): Kohsuke Imai, Takaki Asano, Katsuhiro Arai, Hiroaki Ida, Norimitsu Inoue, Masahiro Ueki, Mariko Eguchi, Satoshi Okada, Nobuo Kanazawa, Naoki Kimura, Toshinao Kawai, Naotomo Kambe, Tomohiro Koga, Masanori Kono, Yoji Sasahara, Sanami Takada, Ichiro Takeuchi, Hiroshi Tsujimoto, Etsushi Toyofuku, Ryusuke Nambu, Junki Hiura, Yukiko Hidaka, Hiroko Fukushima, Takahiko Horiuchi, Yoshitaka Honda, Yusuke Matsuda, Tomoyuki Mizukami, Takako Miyamae, Hideki Muramatsu, Kunihiko Moriya, Takashi Yamazaki, Motoi Yamashita, and Manabu Wakamatsu.
