# Supplementary material for: Clinical characteristics and treatment strategies for A20 haploinsufficiency in Japan: a national epidemiological survey
Source: Front Immunol. 2025 Jun 12;16:1548042. doi: 10.3389/fimmu.2025.1548042 (PMC12197945; doi:10.3389/fimmu.2025.1548042)
Supplement: Supplementary file 1 [file DataSheet1.docx]

Supplementary Material

# Methods

## Genetic analysis

Genomic DNA was extracted from peripheral blood for sequencing of all exons and exon-intron junctions of *TNFAIP3*. Sanger sequencing was performed at Gifu University or in collaborative research institutes of the Primary Immunodeficiency Database in Japan (PIDJ) project. Regarding recently identified cases, next-generation sequencing of target genes related to autoinflammatory disorders, including *TNFAIP3*, was performed at the Kazusa DNA Laboratory (Kisarazu, Japan). Mutation mosaicism was analyzed using the MiSeq Sequencing System (Illumina Inc.,) following PCR amplification of the target, or was based on a previous report (1).

## In vitro functional evaluation of *TNFAIP3* variants

To confirm the pathogenicity of newly identified *TNFAIP3* variants, excluding cases of large deletions and known variants, nuclear factor (NF)-κB-driven luciferase reporter gene assays employing A20-deficient HEK293 cells were performed, as previously described (2). Briefly, the inhibitory effects of the wild-type gene and each variant on nuclear factor (NF)-κB reporter gene activity were compared. The pathogenicity of the truncating variants was examined using an NF-κB-driven luciferase reporter gene activity assay in A20-deficient HEK293 cells stimulated with tumor necrosis factor (TNF)-α (20 ng/mL) for truncating variants or co-transfected with the *CARD11* mutant (F130V), which activates B-cell receptor signaling, for missense variants. The wild-type gene had an inhibitory effect on NF-κB reporter gene activity, while the pathological variants had a disrupted inhibitory effect. *P* values were determined by one-way analysis of variance with Tukey’s multiple comparison test. Patients carrying *TNFAIP3* variants with pathogenicity that could not be confirmed by this functional analysis were excluded from this study. Additionally, previously uncharacterized splice-site variants in *TNFIAP3* were functionally analyzed using the minigene splicing assay.

## Minigene splicing assay

Wild-type (WT) DNA segments of *TNFAIP3* extending from exons 3 to 8 (~ 5.4 kb) and from exons 7 to 9 (~ 4.9 kb) were cloned into plasmid pCAGGS (3) for use as the minigene splicing constructs WTex3-8 and WTex7-9. The *TNFIAP3* splice-site variants c.487-1G>A, c.805+1G>A, c.986+1G>T, and c.2088+1G>A were generated using KOD FX Neo and the KOD Plus Mutagenesis Kit (Toyobo Co., Ltd., Japan). Three of these variants (c.487-1G>A, c.805+1G>A, and c.986+1G>T) were introduced into WTex3-8, and the other (c.2088+1G>A) into WTex7-9. A total of 2.5 µg of each minigene splicing vector was transfected into 5×10^5^ HEK 293T cells (RCB2202, the RIKEN BioResource Research Center, Japan) using Lipofectamine^®︎^ 2000 (Invitrogen, USA). At 48 hours post transfection, mRNA was extracted from the cells and first-strand cDNA was reverse transcribed by M-MLV Reverse Transcriptase (Invitrogen) using oligo (dT) primers. Four first-strand cDNAs (WTex3-8, c.487-1G>A, c.805+1G>A, and c.986+1G>C) were amplified using primers targeted to *TNFAIP3* exons 3 (5'-AGTACATGTGGGGCGTTCAG-3') and 7 (5'-ATGAGAGAAAGCTGGGGCAC-3'), and two first-strand cDNAs (WTex7-9 and c.2088+1G>A) using primers targeted *TNFAIP3* exons 7 (5'-CACCAGCGTTCCAAGTCAGA-3') and 9 (5'-GATGCTGACACTCCATGCAG-3'). PCR-amplified cDNA fragments were separated and visualized on 2% agarose gels to identify potential changes in the splicing process and to provide template for direct sequencing. If necessary, TA cloning was performed before direct sequencing to isolate cDNA fragments.

## Evaluation of type I IFN scores

Expression levels of interferon (IFN)-related genes were measured at Gifu University or Kyoto University (4). Total RNA extracted from human blood samples was collected into PAXgene Blood RNA tubes (762165, Becton, Dickinson and Company, USA) and reverse transcribed by M-MLV Reverse Transcriptase (Invitrogen) using Oligo (dT) primers. Quantitative real-time PCR was performed with TaqPath^TM^ qPCR Master Mix (CG) and TaqMan probes (Thermo Fisher Scientific, USA) on a CFX Opus 96 Real-Time PCR system (Bio-Rad Laboratories, USA). TaqMan probes for *IFI27* (Hs01086370_m1), *IFI44L* (Hs00199115_m1), *IFIT1* (Hs00356631_g1), *ISG15* (Hs00192713_m1), *RSAD2* (Hs01057264_m1), *SIGLEC1* (Hs00988063_ml), and *GAPDH* (Hs02786624_g1) were used. The expression levels of each transcript were determined in triplicate and normalized to the level of *GAPDH*. The IFN score was defined as the median of the relative expression levels of the six IFN-stimulated genes (*IFI27*, *IFI44L*, *IFIT1*, *ISG15*, *RSAD2*, and *SIGLEC1*). An abnormal IFN score was defined as that > two standard deviations from the mean IFN score in the control group.

## Assay of anti-drug antibodies

Anti-drug antibodies to adalimumab, infliximab, etanercept, and tocilizumab were measured using SHIKARI^®︎^ ELISA kits (S-ATA, Q-ATI, S-ATE, and S-ATOC; Matriks Biotek, Turkey), which are enzyme immunoassays for qualitative determination of antibodies to biologics in serum, in accordance with the manufacturer’s instructions.

# Results

## Excluded cases

Twenty cases with missense variants, derived from suspected cases of autoinflammatory disorders with pathogenicity that was not confirmed by our functional analysis, were excluded (**Supplementary Table 6** and **Supplementary Figure 2**). One case with low frequency somatic *TNFAIP3* mosaicism in peripheral blood (c.806-1G>T) was excluded. This is because the transcript analysis of peripheral blood from the patient with 22% somatic mosaicism of c.806-1G>T did not detect any sequence abnormalities, and therefore, mosaic variant c.806-1G>T was considered not to affect the development of A20 haploinsufficiency (HA20). In the other case with low frequency somatic mosaicism (p.Q760X), although functional analysis demonstrated the pathogenicity of the variant (**Figure 2** and **Supplementary Figure 2)**, the case was excluded for reasons described below.

## Low frequency somatic *TNFAIP3* mosaicism

Two individuals with low frequency somatic *TNFAIP3* mosaicism were found in families with HA20 patients and were asymptomatic, with no apparent increase in the type I IFN score and were not considered to have HA20 (**Supplementary Figure 3**). The individual with 14% somatic mosaicism of p.Q760X had Behcet’s disease-like symptoms, such as fever and recurrent stomatitis, but the variant was not thought to be involved in HA20. The reason is that the patient was part of a familial case of Behcet’s disease but two other symptomatic family members did not carry the same variant. However, several autoinflammatory disorders are known to be caused by somatic mosaicism of their responsible genes [5]. Typically, onset due to low frequency somatic mosaicism of *NLRP3* has been reported in cryopyrin-associated periodic syndrome (CAPS) [6], and the in vitro effects of variants, which were found only as somatic *NLRP3* mosaicism, were similar to or more pronounced than those of previously reported heterozygous *NLRP3* variants, suggesting that somatic *NLRP3* mosaicism has pathogenic effects. If the same is true for *TNFAIP3* variants, the results of our in vitro functional analysis using NF-κB reporter gene activity (**Figure 2** and **Supplementary Figure 2**) support the notion that low frequency somatic *TNFAIP3* mosaicism of p.Q760X does not contribute to disease development.

## Results of minigene splicing assay

The direct sequencing of the PCR product from the c.487-1G>A minigene showed a partial deletion of exon 4 (c.487_532del; p.Asn163ThrfsX) (**Supplementary Figure 4B**) and from the c.805+1G>A minigene a skipping of exon 5 (c.635_805del; p.Asp212GlufsX) (**Supplementary Figure 4C**). Two bands were detected on the 2% agarose gel electrophoresis of *TNFAIP3* cDNA derived from the c.986+1G>C variant. Direct sequencing of the smaller PCR product from the c.986+1G>C minigene showed a partial deletion of exon 6 (c.925_986del; p.Ile310GlyfsX) (**Supplementary Figure 4D**). TA cloning prior to direct sequencing of the larger PCR product from the c.986+1G>C minigene to isolate cDNA fragments. Direct sequencing showed partial insertion of three patterns of intron 6 (insertion of g.10070_10164, g.10070_10218, and g.10070_10233; p.Lys329AsnfsX) (**Supplementary Figure 4D**). Direct sequencing of the PCR product from the c.2088+1G>A minigene showed a skipping of exon 8 (c.1906_2097del; p.His636GlufsX) (**Supplementary Figure 4E**).

# References

1. Tozaki N, Tawada C, Niwa H, Mizutani Y, Shu E, Kawase A, et al. A case of VEXAS syndrome (vacuoles, E1 enzyme, X-linked, autoinflammatory, somatic) with decreased oxidative stress levels after oral prednisone and tocilizumab treatment. Front Med (Lausanne). 2022;9:1046820. doi: 10.3389/fmed.2022.1046820

2. Kadowaki S, Hashimoto K, Nishimura T, Kashimada K, Kadowaki T, Kawamoto N, et al. Functional analysis of novel A20 variants in patients with atypical inflammatory diseases. Arthritis Res Ther. 2021;23(1):52. doi: 10.1186/s13075-021-02434-w

3. Niwa H, Yamamura K, Miyazaki J. Efficient selection for high-expression transfectants with a novel eukaryotic vector. Gene. 1991;108(2):193-9. doi: 10.1016/0378-1119(91)90434-d

4. Miyamoto T, Honda Y, Izawa K, Kanazawa N, Kadowaki S, Ohnishi H, et al. Assessment of type I interferon signatures in undifferentiated inflammatory diseases: A Japanese multicenter experience. Front Immunol. 2022;13:905960. doi: 10.3389/fimmu.2022.905960

5. Aluri J, Cooper MA. Somatic mosaicism in inborn errors of immunity: Current knowledge, challenges, and future perspectives. Semin Immunol. 2023;67:101761. doi: 10.1016/j.smim.2023.101761

6. Tanaka N, Izawa K, Saito MK, Sakuma M, Oshima K, Ohara O, et al. High incidence of NLRP3 somatic mosaicism in patients with chronic infantile neurologic, cutaneous, articular syndrome: results of an International Multicenter Collaborative Study. Arthritis Rheum. 2011;63(11):3625-32. doi: 10.1002/art.30512

7. Kadowaki T, Ohnishi H, Kawamoto N, Hori T, Nishimura K, Kobayashi C, et al. Haploinsufficiency of A20 causes autoinflammatory and autoimmune disorders. J Allergy Clin Immunol. 2018;141(4):1485-8 e11. doi: 10.1016/j.jaci.2017.10.039

8. Ohnishi H, Kawamoto N, Seishima M, Ohara O, Fukao T. A Japanese family case with juvenile onset Behcet's disease caused by TNFAIP3 mutation. Allergol Int. 2017;66(1):146-8. doi: 10.1016/j.alit.2016.06.006

9. Shiraki M, Williams E, Yokoyama N, Shinoda K, Nademi Z, Matsumoto K, et al. Hematopoietic Cell Transplantation Ameliorates Autoinflammation in A20 Haploinsufficiency. J Clin Immunol. 2021;41(8):1954-6. doi: 10.1007/s10875-021-01124-1

10. Hori T, Ohnishi H, Kadowaki T, Kawamoto N, Matsumoto H, Ohara O, et al. Autosomal dominant Hashimoto's thyroiditis with a mutation in TNFAIP3. Clin Pediatr Endocrinol. 2019;28(3):91-6. doi: 10.1297/cpe.28.91

11. Iwasa T, Miwa T, Unome S, Hanai T, Imai K, Takai K, et al. A case of A20 haploinsufficiency complicated by autoimmune hepatitis. Hepatol Res. 2024;54(6):606-11. doi: 10.1111/hepr.14003

12. Shigemura T, Kaneko N, Kobayashi N, Kobayashi K, Takeuchi Y, Nakano N, et al. Novel heterozygous C243Y A20/TNFAIP3 gene mutation is responsible for chronic inflammation in autosomal-dominant Behcet's disease. RMD Open. 2016;2(1):e000223. doi: 10.1136/rmdopen-2015-000223

13. Mitsunaga K, Inoue Y, Naito C, Ogata H, Itoh Y, Natsui Y, et al. A case of A20 haploinsufficiency in which intestinal inflammation improved with thalidomide. Rheumatology (Oxford). 2023;62(6):e193-e5. doi: 10.1093/rheumatology/keac634

14. Takagi M, Ogata S, Ueno H, Yoshida K, Yeh T, Hoshino A, et al. Haploinsufficiency of TNFAIP3 (A20) by germline mutation is involved in autoimmune lymphoproliferative syndrome. J Allergy Clin Immunol. 2017;139(6):1914-22. doi: 10.1016/j.jaci.2016.09.038

15. Sato S, Fujita Y, Shigemura T, Matoba H, Agematsu K, Sumichika Y, et al. Juvenile onset autoinflammatory disease due to a novel mutation in TNFAIP3 (A20). Arthritis Res Ther. 2018;20(1):274. doi: 10.1186/s13075-018-1766-x

16. Shimizu M, Matsubayashi T, Ohnishi H, Nakama M, Izawa K, Honda Y, et al. Haploinsufficiency of A20 with a novel mutation of deletion of exons 2-3 of TNFAIP3. Mod Rheumatol. 2021;31(2):493-7. doi: 10.1080/14397595.2020.1719595

17. Tsuchida N, Kirino Y, Soejima Y, Onodera M, Arai K, Tamura E, et al. Haploinsufficiency of A20 caused by a novel nonsense variant or entire deletion of TNFAIP3 is clinically distinct from Behcet's disease. Arthritis Res Ther. 2019;21(1):137. doi: 10.1186/s13075-019-1928-5

18. Taniguchi K, Inoue M, Arai K, Uchida K, Migita O, Akemoto Y, et al. Novel TNFAIP3 microdeletion in a girl with infantile-onset inflammatory bowel disease complicated by a severe perianal lesion. Hum Genome Var. 2021;8(1):1. doi: 10.1038/s41439-020-00128-4

19. Uchida T, Suzuki T, Kikuchi A, Kakuta F, Ishige T, Nakayama Y, et al. Comprehensive Targeted Sequencing Identifies Monogenic Disorders in Patients With Early-onset Refractory Diarrhea. J Pediatr Gastroenterol Nutr. 2020;71(3):333-9. doi: 10.1097/MPG.0000000000002796

20. Endo Y, Funakoshi Y, Koga T, Furukawa K, Sasaki D, Miura K, et al. Paediatric-onset haploinsufficiency of A20 associated with a novel and de novo nonsense TNFAIP3 mutation. Rheumatology (Oxford). 2020;59(11):e85-e7. doi: 10.1093/rheumatology/keaa206

21. Endo Y, Funakoshi Y, Koga T, Ohashi H, Takao M, Miura K, et al. Large deletion in 6q containing the TNFAIP3 gene associated with autoimmune lymphoproliferative syndrome. Clin Immunol. 2022;235:108853. doi: 10.1016/j.clim.2021.108853

22. Imai T, Shiraishi A, Nishiyama K, Ishimura M, Ohga S. Lipopolysaccharide-induced monocyte death in a novel ZnF7 domain mutation of TNFAIP3. J Allergy Clin Immunol Pract. 2020;8(6):2071-4 e5. doi: 10.1016/j.jaip.2020.01.026

23. Shirai H, Saito-Sato N, Horiuchi E, Kikuchi H, Kadowaki S, Ohnishi H, et al. Case report: Adult case of A20 haploinsufficiency suspected as neuro-Behçet disease. Front Immunol. 2024;15:1508307. doi: 10.3389/fimmu.2024.1508307.

24. Wakatsuki R, Hatai Y, Okamoto K, Kaneko S, Shimbo A, Irabu H, et al. An infant with A20 haploinsufficiency presenting with periodic fever syndrome: A case report. Int J Rheum Dis. 2023;26(5):973-6. doi: 10.1111/1756-185X.14564

# Figure legends

**Supplementary Figure 1.** Pedigrees of 37 families with heterozygous *TNFAIP3* variants. Hatched cases indicate those with somatic mosaicism of *TNFAIP3*. *Non-analyzed genetically predisposed HA20 patients; ^†^Asymptomatic carrier of heterozygous *TNFAIP3* variants; ^‡^Non-analyzed genetically predisposed asymptomatic carrier of heterozygous *TNFAIP3* variants.

**Supplementary Figure 2.** Reporter gene activity of missense variants and Q760X of *TNFAIP3*. The inhibitory effects of the wild-type gene and each variant on nuclear factor (NF)-κB reporter gene activity were compared. Activities of missense variants **(A)** and Q760X **(B)** of *TNFAIP3*. For missense variants, *TNFAIP3* variant activities were measured using an NF-κB luciferase reporter gene assay in A20-deficient HEK293 cells co-transfected with the *CARD11* mutant (F130V), which activates B-cell receptor signaling, instead of tumor necrosis factor (TNF)-α stimulation, which was the evaluation method of truncating variants. The wild-type gene had an inhibitory effect on NF-κB reporter gene activity, while the pathological variants had a disrupted inhibitory effect. For Q760X, because pathogenicity was not demonstrated using an NF-κB-driven luciferase reporter gene activity assay enhanced by stimulation with TNF-α, pathogenicity was evaluated using F130V co-transfection; *P < 0.05, **P < 0.01, ***P < 0.001, ****P < 0.0001, as determined by one-way analysis of variance with Tukey’s multiple comparison test. RLU, relative light unit.

**Supplementary Figure 3.** Interferon **(**IFN) scores by variant type. IFN scores measured in healthy control patients and three types of variants at Gifu university. Red spots indicate IFN scores of ≥ 5.11, based on two standard deviations from the mean score of healthy controls; *P < 0.05, **P < 0.01, ***P <0 .001, ****P <0 .0001, as determined by one-way analysis of variance with Tukey’s multiple comparison test.

**Supplementary Figure 4.** Minigene splicing assay. (**A)** 2% agarose gel electrophoresis of *TNFAIP3* cDNA derived from wild-type with exons 3-8 and three variant minigenes (c.487-1G>A, c.805+1G>A, and c.986+1G>C). Sanger sequencing of cDNA fragments from the variant minigenes: **(B)** c.487-1G>A, **(C)** c.805+1G>A, and **(D)** c.986+1G>C. **(E)** 2% agarose gel electrophoresis of *TNFAIP3* cDNA derived from wild-type with exon 7-9 and the variant minigene c.2088+1G>A, and Sanger sequencing of cDNA fragments from the variant minigene.

**
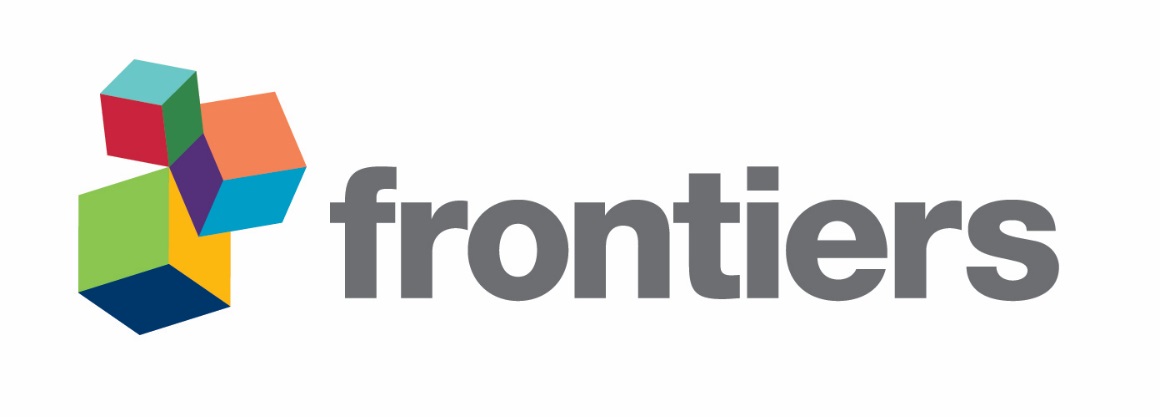
**
