# Supplementary material for: Clinical characteristics and treatment strategies for A20 haploinsufficiency in Japan: a national epidemiological survey
Source: Front Immunol. 2025 Jun 12;16:1548042. doi: 10.3389/fimmu.2025.1548042 (PMC12197945; doi:10.3389/fimmu.2025.1548042)
Supplement: Supplementary file 2 [file DataSheet2.docx]

**Supplementary Table 1.** Characteristics and treatments of 54 patients with A20 haploinsufficiency in Japan

| Family no. | Patient no. | Sex | Age at onset | Genotype | Previous/ surgical treatment* | Current treatment | reference |
| --- | --- | --- | --- | --- | --- | --- | --- |
| F1 | **P1** | F | 10 yr | p.W85GfsX | NSAIDs, colchicine, MTX, TCZ | PSL, IGU, ADA | (7,8) |
|  | **P2** | F | 9 mo | p.W85GfsX | Colchicine | PSL | (7,8) |
| F2 | **P3** | M | 10 mo | c.2088+5G>C | NSAIDs, colchicine, mesalazine, CS, MTX, CyA, TCZ, ADA, ETN, IFX, TOF, CAN, GLM, HCT | - | (7,9) |
| F3 | **P4** | F | 6 mo | p.Q737SfsX | Colchicine, PSL, ETN | ADA | (7,10) |
|  | **P5** | F | 2 yr | p.Q737SfsX | - | Colchicine |  |
|  | **P6** | F | 5 yr | p.Q737SfsX | Colchicine | PSL, AZA | (7,11) |
|  | **P7** | F | Early  childhood | p.Q737SfsX | Colchicine | Unknown | (7) |
|  | **P8** | M | Unknown | p.Q737SfsX | Craniopharyngioma removal | - | (7) |
|  | **P9** | F | 1 yr | p.Q737SfsX | - | Colchicine | (7) |
|  | **P10** | F | 2 mo | p.Q737SfsX | - | Colchicine | (7) |
| F4 | **P11** | M | 3 yr | c.1906+1G>A | PSL, cimetidine, tonsillectomy | MTX, ADA | (7) |
|  | **P12** | M | Childhood | c.1906+1G>A | - | - | (7) |
| F5 | **P13** | M | 17 yr | p.C243Y | - | Colchicine, PSL | (7,12) |
|  | **P14** | F | 17 yr | p.C243Y | - | Colchicine, PSL | (7,12) |
| F6 | **P15** | F | 1 yr | p.N449TfsX | PSL | Colchicine, mesalazine | (7) |
| F7 | **P16** | M | 0 mo | p.A588VfsX | Mesalazine, CS, IFX, ETN, GLM, enterectomy, endoscopic balloon dilation | Colchicine, PSL, thalidomide, MTX, ADA | (7,13) |
|  | **P17** | M | 20 yr | p.A588VfsX | - | - | (7) |
| F8 | **P18** | M | 1 yr | p.K417SfsX | IVIG, CS, CyA, MMF, TAC, MTX, ADA, IFX, TOF | PSL, ADA | (7,14) |
| F9 | **P19** | M | 1 yr | p.R45X | Pranlukast | Colchicine, PSL | (4,7) |
| F10 | **P20** | M | 11 mo | p.E192K | Cimetidine, PSL, tonsillectomy | - | (2) |
|  | **P21** | M | unknown | p.E192K | - | - |  |
| F11 | **P22** | M | 0 m | p.C200AfsX | PSL | Unknown | (15) |
|  | **P23** | F | 4 yr | p.C200AfsX | - | Unknown | (15) |
| F12 | **P24** | M | 6 yr | Exon 2-3 deletion | Colchicine, AZA, IFX | Mesalazine, MTX, ADA | (16) |
| F13 | **P25** | F | 12 yr | p.C478X | Cimetidine, PSL | Colchicine | (17) |
|  | **P26** | M | Infancy | p.C478X | Colchicine | PSL, ADA | (17) |
| F14 | **P27** | F | 2 mo | 236-kb deletion  at 6q23.3 | NSAIDs, mesalazine, PSL, MTX, ADA | Colchicine, cimetidine | (17) |
| F15 | **P28** | F | 7 mo | 119-kb  microdeletion | Mesalazine, ADA | Colchicine, PSL, MTX, UST | (18) |
| F16 | **P29** | M | 11 yr | p.R204EfsX | NSAIDs, MTX, enterectomy | Colchicine, PSL, thalidomide, IFX | (19) |
| F17 | **P30** | F | 11 mo | c.487-1G>A | - | - | (19) |
| F18 | **P31** | F | 1 yr | p.S408X | PSL | Colchicine, IFX | (20) |
| F19 | **P32** | F | 3 yr | c.805+1G>A | - | Colchicine |  |
|  | **P33** | F | 10 yr | c.805+1G>A | PSL, mesalazine | - |  |
|  | **P34** | F | 10 yr | c.805+1G>A | PSL, IFX | Colchicine |  |
|  | **P35** | F | 2 yr | c.805+1G>A | Mesalazine, MTX | PSL, ADA |  |
| F20 | **P36** | M | 3 yr | p.R45X | - | Colchicine, mesalazine | (4) |
| F21 | **P37** | F | 7 mo | p.G583X | - | Colchicine | (4) |
| F22 | **P38** | F | 6 mo | 3.3-Mb deletion  at 6q23.3 | CS | MMF | (21) |
| F23 | **P39** | F | 7 yr | p.Q187X | CS, TAC, IFX, ADA | MMF, TOF | (22) |
| F24 | **P40** | F | 3 d | p.K759fs | PSL | ADA | (22) |
| F25 | **P41** | M | 10 yr | p.C243Y | CS, Colchicine, Mesalazine | ADA |  |
| F26 | **P42** | M | 14 yr | p.M174fs | TAC | Colchicine, MTX |  |
| F27 | **P43** | M | 3 mo | p.A773fsX | CS, cimetidine | Colchicine |  |
|  | **P44** | M | 27 yr | p.A773fsX | CS | Colchicine, PSL, ADA |  |
| F28 | **P45** | F | 4 yr | p.L324QsfsX | NSAIDs, colchicine, mesalazine | MTX, ADA |  |
| F29 | **P46** | F | 5 yr | p.S507AfsX |  | Colchicine, PSL, IGU |  |
| F30 | **P47** | M | 17 yr | p.R87X | Colchicine | PSL, MTX, ADA | (23) |
| F31 | **P48** | F | 6 mo | p.P226LfsX | CS, IFX | Colchicine, AZA, ADA | (24) |
| F32 | **P49** | M | 10 d | c.2088+1G>A | Colchicine, cimetidine, CyA, | PSL |  |
| F33 | **P50** | F | 10 yr | c.986+1G>C | CS, colchicine, ANI, tonsillectomy | PSL, HDQ | (4) |
| F34 | **P51** | M | 7 yr | p.E751X | - | Colchicine, PSL |  |
| F35 | **P52** | F | 3 yr | p.R271X | NSAIDs, PSL, cimetidine, MTX, ADA, TCZ, IFX | colchicine, AZA, GLM |  |
| F36 | **P53** | M | 10 mo | p.C612WfsX | AZA, IFX | PSL, ADA |  |
| F37 | **P54** | M | 8 yr | 2.8-Mb deletion  at q.23.3-24.1 | PSL | Mesalazine |  |

*CSs used in previous treatments included prednisolone, intravenous methylprednisolone, dexamethasone, dexamethasone palmitate, and budesonide. ADA, adalimumab; ANI, anifrolumab; AZA, azathioprine; CAN, canakinumab; CS, corticosteroid; CyA, cyclosporin A; ETN, etanercept; GLM, golimumab; HCT, hematopoietic cell transplantation; HDQ, hydroxychloroquine; IFX, infliximab; IGU, iguratimod; IVIG, intravenous immunoglobulin; MMF, mycophenolate mofetil; MTX, methotrexate; NSAIDs, non-steroidal anti-inflammatory drugs; PSL, prednisolone; TAC, tacrolimus; TCZ, tocilizumab; TOF, tofacitinib; UST, ustekinumab.

**Supplementary Table 2.** Clinical features of 54 patients with A20 haploinsufficiency

| Patient No. | Initial diagnosis | Recurrent  fever | Ulcer  (oral/  genital) | Intestinal  symptoms | Musculoskeletal  symptoms | Cutaneous  symptoms | Autoimmune  disease | Others/ medical history | IFN score |
| --- | --- | --- | --- | --- | --- | --- | --- | --- | --- |
| P1 | RF negative poly JIA→intestinal BD | +  Paroxysmal | +/+ | + | Poly arthritis | AD | − | Eyelid conjunctival aphthae  Recurrent tonsillitis | Elevated  (4) |
| P2 | PFAPA-like recurrent fever and stomatitis→  intestinal BD | + | +/+ | + | − | − | − | − |  |
| P3 | Systemic JIA→psoriatic arthritis | +  Paroxysmal  / prolonged | −/+ | + | Poly arthritis, spondyloarthritis, pharyngitis | Psoriasis | Psoriatic arthritis | Aortic regurgitation,  Aseptic pyuria | Elevated  (4) |
| P4 | FMF-like autoinflammatory disease | +  Paroxysmal | −/− | + | − | AD | − | -− | Elevated  (4) |
| P5 | − | - | −/− | + | − | − | HD | Lymphadenitis, Liver dysfunction | Elevated |
| P6 | Crohn’s disease,  HD | +  Paroxysmal  / prolonged | +/+ | + | − | − | HD, AIH | Lymphadenopathy | Elevated  (4) |
| P7 | HD | + | +/− | + | − | − | HD | -− | Elevated  (4) |
| P8 | Hodgkin’s lymphoma, craniopharyngioma, HD | - | +/− | + | − | − | HD | Hodgkin’s lymphoma,  craniopharyngioma,  Elevated biliary enzymes |  |
| P9 | − | +  Paroxysmal | +/− | − | − | − | HD | Liver dysfunction | Elevated |
| P10 | − | +  Paroxysmal  / prolonged | +/− | − | − | − | − | Aseptic meningitis (elevated IL-6 in spinal fluid), unidentified pneumonia with nodular and granular shadow on chest CT,  BCG dermatitis, cheilitis | Elevated |
| P11 | RF-negative poly JIA→PFAPA | +  Paroxysmal | +/− | − | Poly arthritis,  tenosynovitis | − | − | / PFAPA (suspected) |  |
| P12 | - | - | +/+ | + | − | − | − | / IgA vasculitis |  |
| P13 | Incomplete BD | +  Paroxysmal  / prolonged | +/− | − | − | Acneiform lesions, EN | − | / Nephrotic syndrome | Normal |
| P14 | BD | +  Prolonged | +/+ | + | − | EN | − | − | Elevated |
| P15 | PFAPA | + | +/− | + | − | EN | − | − |  |
| P16 | IBD | +  Paroxysmal | +/− | + | − | − | − | Infectious mononucleosis,  Persistent EBV DNA in peripheral blood |  |
| P17 | − | +  Paroxysmal | +/+ | − | − | Acneiform lesions | Graves’ disease | − |  |
| P18 | ALPS-like disease | +  Paroxysmal | +/+ | + | Poly arthritis | Toxic skin eruption | AIH | Kawasaki disease,  Nephrotic syndrome | Elevated |
| P19 | PFAPA | +  Paroxysmal | +/+ | + | − | − | − | − | Elevated  (4) |
| P20 | PFAPA | +  Paroxysmal  / prolonged | −/− | − | Transient femoral arthritis | − | − | − | Normal |
| P21 | − | - | −/− | + | − | Acneiform lesions, AD | − | Floaters, Flash scotoma | Normal |
| P22 | BD susp | + | +/− | + | − | − | − | Headache |  |
| P23 | BD | - | +/+ | + | − | − | − | − |  |
| P24 | Intestinal BD | +  Paroxysmal | +/− | + | − | − | − | Headache | Elevated |
| P25 | BD | +  Paroxysmal | +/+ | + | Arthralgia | EN | − | Dry eye |  |
| P26 | BD | + | +/+ | + | Arthralgia | Acneiform lesions | − | Myocarditis, lightheadedness,  adjustment disorder,  lymphadenopathy |  |
| P27 | BD | +  Paroxysmal | +/− | + | Arthralgia, stiffness | Erythema multiforme | HD | Uveitis, headache, core ataxia,  hand tremor |  |
| P28 | BD, Crohn’s disease | +  Paroxysmal | +/+ | + | Poly arthritis | EN | − | − |  |
| P29 | JIA, intestinal BD | +  Paroxysmal | +/+ | + | Arthralgia | EN | − | − |  |
| P30 | Fever of unknown origin | + | −/− | + | − | Erythema | − | − |  |
| P31 | Acute bronchitis, chronic sinusitis, purulent lymphadenitis | +  Paroxysmal  / prolonged | +/− | + | Arthralgia | − | − | Lymphadenopathy |  |
| P32 | BD | +  Paroxysmal | +/+ | − | − | − | − | − |  |
| P33 | BD | +  Paroxysmal | +/+ | + | Poly arthritis | Acneiform lesions | − | Meningitis |  |
| P34 | BD | +  Paroxysmal | +/+ | + | Arthralgia | − | SLE | − |  |
| P35 | BD | +  Paroxysmal | +/− | + | − | − | − | Headache,  psychiatric symptoms |  |
| P36 | IBD | +  Paroxysmal | +/− | + | Arthralgia | Urticaria | HD | / IgA vasculitis | Elevated  (4) |
| P37 | Periodic fever  (HIDS, PFAPA) | +  Paroxysmal | +/− | + | Poly arthritis | Purple macular rash | − | − | Elevated  (4) |
| P38 | ALPS | + | −/− | + | − | Livedo reticularis | AIHA, ITP | Acute encephalopathy (seizures, impaired consciousness),  cervical lymphadenitis |  |
| P39 | IgA vasculitis→SLE, SS | - | +/− | + | − | Acneiform lesions | SLE, SS | Lupus nephritis |  |
| P40 | Intestinal BD, CAPS susp | +  Paroxysmal | +/− | + | − | Acneiform lesions | − | − |  |
| P41 | Chronic hepatitis | +  Paroxysmal | +/− | + | − | − | − | Chronic hepatitis | Elevated |
| P42 | Tonsilitis | +  Paroxysmal | +/+ | − | Arthralgia | − | − | − |  |
| P43 | PFAPA | +  Paroxysmal  / prolonged | +/− | − | − | − | − | Lymphadenopathy/HLH |  |
| P44 | Juvenile lacunar infarction with leukoencephalopathy | +  Paroxysmal | −/− | − | − | − | − | Lacunar infarction with leukoencephalopathy (dysarthria and paralysis), seizures |  |
| P45 | JIA (enthesitis-associated arthritis) | +  Paroxysmal | −/− | + | Oligo arthritis, enthesitis | − | − | − |  |
| P46 | Crohn’s disease susp | +  Paroxysmal | −/− | + | − | − | − | − |  |
| P47 | Neurological BD | +  Paroxysmal  / prolonged | +/+ | − | Arthralgia, joint swelling | Acneiform lesions | − | Impaired vision, diplopia, and hiccups with cerebral matter lesions on head MRI (elevated IL-6 in spinal fluid),  sensorineural hearing loss, granular shadow with consolidation and bronchiectasis on chest CT, epididymitis |  |
| P48 | Periodic fever syndrome | +  Paroxysmal | −/− | + | − | Erythema | − | − |  |
| P49 | Cytophagic histiocytic panniculitis | +  Paroxysmal | +/− | + | − | EN | − | − |  |
| P50 | SLE, SS→TRAPS | +  Prolonged | +/+ | − | − | EN, Photosensitivity | SLE, SS | Subacute necrotizing lymphadenitis | Elevated  (4) |
| P51 | Subacute necrotizing lymphadenitis | +  Prolonged | +/− | + | − | − | − | Subacute necrotizing lymphadenitis, liver dysfunction |  |
| P52 | Oligo-JIA | +  Paroxysmal | +/− | + | Oligo arthritis | − | − | − |  |
| P53 | Type 1 diabetes mellitus | - | −/− | − | − | Acneiform lesions | Type 1 diabetes mellitus, AIH susp, HD | − |  |
| P54 | Crohn’s disease | - | +/− | + | Arthralgia | − | − | Posterior iris adhesions, multiple small nodules on chest CT |  |

AD, atopic dermatitis; AIH, autoimmune hepatitis; AIHA, autoimmune hemolytic anemia; ALPS; autoimmune lymphoproliferative syndrome; BCG, bacille Calmette-Guerin; BD, Behcet's disease; CAPS, cryopyrin-associated periodic syndrome; CT, computed tomography; EBV, Epstein-Barr virus; EN, erythema nodosum; FMF, familial Mediterranean fever; IBD, inflammatory bowel disease; IL, interleukin; ITP, immune thrombocytopenia; HS, Hashimoto’s disease; HIDS, hyper IgD syndrome; HLH, hemophagocytic lymphohistiocytosis; JIA, juvenile idiopathic arthritis; PFAPA, periodic fever, aphthous stomatitis, pharyngitis and adenitis; RF, rheumatoid factor; SLE, systemic lupus erythematosus; SS, Sjogren’s syndrome; Susp, suspected; TRAPS, tumor necrosis factor receptor-associated periodic syndrome.

**Supplementary Table 3.** Results of assays of anti-drug antibodies to biologic drugs

| **Biologic** | **Patient**  **No** | **Absorbance** | **Result*** | **Effectiveness of biologic**  **with timing of measurement** |
| --- | --- | --- | --- | --- |
| ADA | P1 | 0.046 | Negative | Effective with increased ADA |
|  | P3 | 3.193 | Positive | Timing coincided with secondary failure |
|  | P3 | 0.068 | Negative | After discontinuation of ADA |
|  | P4 | 0.047 | Negative | Effective and in use |
|  | P11 | 0.056 | Negative | Effective and in use |
|  | P16 | 0.016 | Negative | Timing coincided with secondary failure |
|  | P18 | 0.061 | Negative | After discontinuation of ADA |
|  | P24 | 0.334 | Positive | Effective with increased ADA |
|  | P45 | 0.144 | Positive | Effective and in use |
|  | P48 | 0.44 | Negative | Effective and in use |
|  | P52 | 0.056 | Negative | Timing coincided with secondary failure |
| IFX | P3 | 0.006 | Negative | Timing coincided with secondary failure |
|  | P16 | 0.006 | Negative | After discontinuation of IFX |
|  | P18 | 0.03 | Negative | After discontinuation of IFX |
|  | P24 | 0.023 | Negative | Timing coincided with secondary failure |
|  | P24 | 3.985 | Positive | At the onset of infusion reaction |
|  | P24 | 0.053 | Positive | After discontinuation of IFX |
|  | P48 | 0.026 | Negative | After discontinuation of IFX |
|  | P52 | 0.022 | Negative | Timing coincided with reduction of IFX efficacy |
|  | P53 | 3.94 | Positive | At the onset of infusion reaction |
| ETN | P3 | 0.015 | Negative | Timing coincided with reduction of ETN efficacy |
|  | P4 | 0.012 | Negative | Timing coincided with secondary failure |
|  | P16 | 0.022 | Negative | After discontinuation of ETN |
| TCZ | P1 | 0.009 | Negative | Timing coincided with secondary failure |
|  | P3 | 0.01 | Negative | After discontinuation of TCZ |
|  | P52 | 0.014 | Negative | Timing coincided with primary failure |

*Positivity was indicated by an absorbance (450/650nm) of > 0.102, 0.036, 0.028, and 0.041 for adalimumab (ADA), infliximab (IFX), etanercept (ETN), and tocilizumab (TCZ), respectively.

**Supplementary Table 4.** The rate of concomitant immunosuppressant use with molecular target drugs (MTDs)

|  | **MTDs with**  **secondary failure (n)** | **MTDs without**  **secondary failure (n)** |
| --- | --- | --- |
| With concomitant use  of immunosuppressants | 6 (60%) | 19 (52.8%) |
| Without concomitant use  of immunosuppressants | 4 (40%) | 17 (47.2%) |

**Supplementary Table 5.** Human leukocyte antigen (HLA) type by severity of A20 haploinsufficiency

| **HLA type** | **Mild cases* (n)** | **Severe cases* (n)** | **Intractable cases* (n)** |
| --- | --- | --- | --- |
| A26 | 3 / 14 | 1 / 7 | 1 / 10 |
| B27 | 0 / 14 | 0 / 7 | 1 / 10 |
| B51 | 2 / 14 | 1 / 7 | 3 / 10 |
| B52 | 0 / 14 | 2 / 7 | 2 / 10 |

*Mild, severe, and intractable cases were defined as cases in which molecular target drugs (MTDs) were not used, cases in which MTDs were used (excluding intractable cases), and cases in which a change in the initial MTD was required, respectively.

**Supplementary Table 6.** Missense *TNFAIP3* variants excluded by functional analysis

| **Genotype, DNA / Protein** |
| --- |
| c.229C>A / p.L77M |
| c.301G>A / p.G101S |
| c.899A>C / p.G300A |
| c.929T>C / p.I310T |
| c.1759+8C>T / p.E361K |
| c.1129G>A / p.V377M |
| c.1306G>A / p.G436R |
| c.1504C>T / p.R502W |
| c.1520G>A / p.S507N |
| c.1748_1749delinsCG / p.G583A |
| c.1897G>C / p.E633Q |
| c.2093C>T / p.S698L |
| c.2117G>A / p.R706Q |
| c.2126A>G / p.Q709R |
| c.2140C>T / p.P714S |
| c.2144A>C / p.K715T |
