# Supplementary material for: Clinical characteristics and treatment strategies for A20 haploinsufficiency in Japan: a national epidemiological survey
Source: Front Immunol. 2025 Jun 12;16:1548042. doi: 10.3389/fimmu.2025.1548042 (PMC12197945; doi:10.3389/fimmu.2025.1548042)
Supplement: Supplementary file 3 [file Image1.pdf]

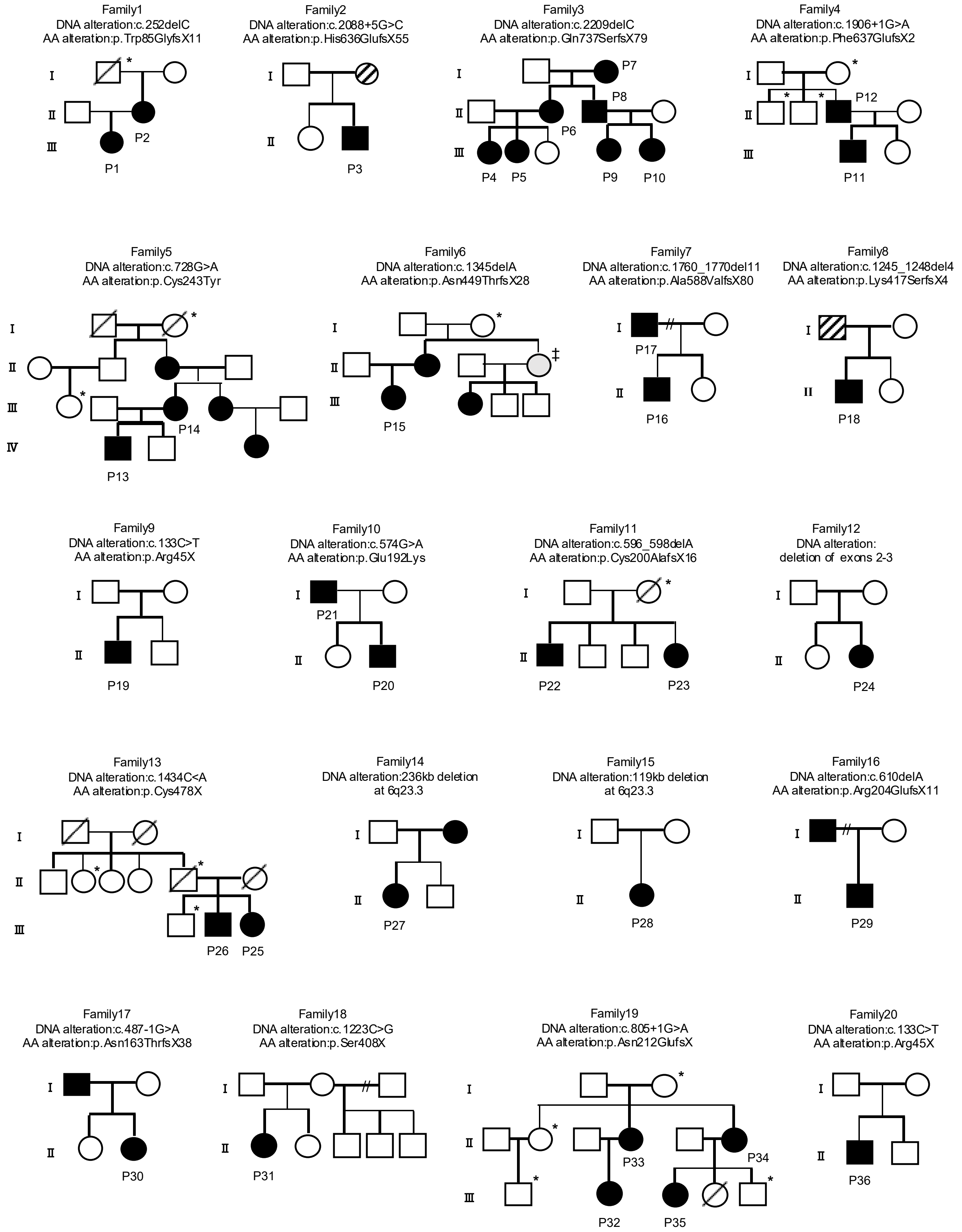

Family1
DNA alteration:c.252delC
AA alteration:p.Trp85GlyfsX11
P1
P2
Family2
DNA alteration:c.2088+5G>C
AA alteration:p.His636GlufsX55
P3
Family3
DNA alteration:c.2209delC
AA alteration:p.Gln737SerfsX79
P4
P5
P6
P7
P8
P9
P10
Family4
DNA alteration:c.1906+1G>A
AA alteration:p.Phe637GlufsX2
P11
P12
Family5
DNA alteration:c.728G>A
AA alteration:p.Cys243Tyr
P13
P14
Family6
DNA alteration:c.1345delA
AA alteration:p.Asn449ThrfsX28
P15
Family7
DNA alteration:c.1760_1770del11
AA alteration:p.Ala588ValfsX80
P16
P17
Family8
DNA alteration:c.1245_1248del4
AA alteration:p.Lys417SerfsX4
P18
Family9
DNA alteration:c.133C>T
AA alteration:p.Arg45X
P19
Family10
DNA alteration:c.574G>A
AA alteration:p.Glu192Lys
P20
P21
Family11
DNA alteration:c.596_598delA
AA alteration:p.Cys200AlafsX16
P22
P23
Family12
DNA alteration:
deletion of exons 2-3
P24
Family13
DNA alteration:c.1434C<A
AA alteration:p.Cys478X
P25
P26
Family14
DNA alteration:236kb deletion
at 6q23.3
P27
Family15
DNA alteration:119kb deletion
at 6q23.3
P28
Family16
DNA alteration:c.610delA
AA alteration:p.Arg204GlufsX11
P29
Family17
DNA alteration:c.487-1G>A
AA alteration:p.Asn163ThrfsX38
P30
Family18
DNA alteration:c.1223C>G
AA alteration:p.Ser408X
P31
Family19
DNA alteration:c.805+1G>A
AA alteration:p.Asn212GlufsX
P32
P33
P34
P35
Family20
DNA alteration:c.133C>T
AA alteration:p.Arg45X
P36

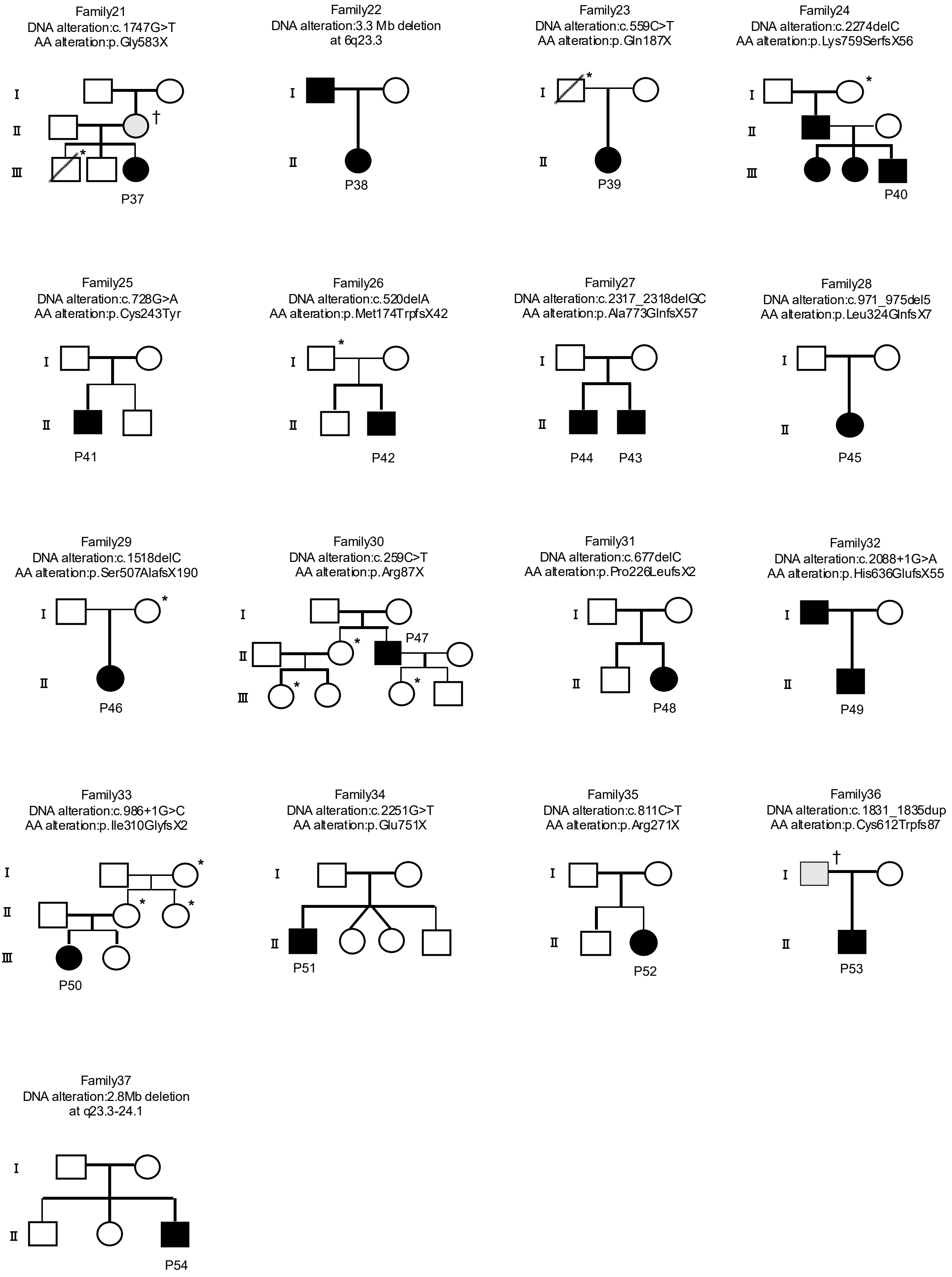

Family21
DNA alteration:c.1747G>T
AA alteration:p.Gly583X
I
II
III
*
†
P37
Family22
DNA alteration:3.3 Mb deletion
at 6q23.3
I
II
P38
Family23
DNA alteration:c.559C>T
AA alteration:p.Gln187X
*
I
II
P39
Family24
DNA alteration:c.2274delC
AA alteration:p.Lys759SerfsX56
I
II
III
*
P40
Family25
DNA alteration:c.728G>A
AA alteration:p.Cys243Tyr
I
II
P41
Family26
DNA alteration:c.520delA
AA alteration:p.Met174TrpfsX42
*
I
II
P42
Family27
DNA alteration:c.2317_2318delGC
AA alteration:p.Ala773GlnfsX57
I
II
P44
P43
Family28
DNA alteration:c.971_975del5
AA alteration:p.Leu324GlnfsX7
I
II
P45
Family29
DNA alteration:c.1518delC
AA alteration:p.Ser507AlafsX190
I
II
*
P46
Family30
DNA alteration:c.259C>T
AA alteration:p.Arg87X
I
II
III
*
P47
*
*
Family31
DNA alteration:c.677delC
AA alteration:p.Pro226LeufsX2
I
II
P48
Family32
DNA alteration:c.2088+1G>A
AA alteration:p.His636GlufsX55
I
II
P49
Family33
DNA alteration:c.986+1G>C
AA alteration:p.Ile310GlyfsX2
I
II
III
*
*
*
P50
Family34
DNA alteration:c.2251G>T
AA alteration:p.Glu751X
I
II
P51
Family35
DNA alteration:c.811C>T
AA alteration:p.Arg271X
I
II
P52
Family36
DNA alteration:c.1831_1835dup
AA alteration:p.Cys612Trpfs87
†
I
II
P53
Family37
DNA alteration:2.8Mb deletion
at q23.3-24.1
I
II
P54
