# Supplementary figures and images for: Clinical characteristics and treatment strategies for A20 haploinsufficiency in Japan: a national epidemiological survey
Source: Front Immunol. 2025 Jun 12;16:1548042. doi: 10.3389/fimmu.2025.1548042 (PMC12197945; doi:10.3389/fimmu.2025.1548042)

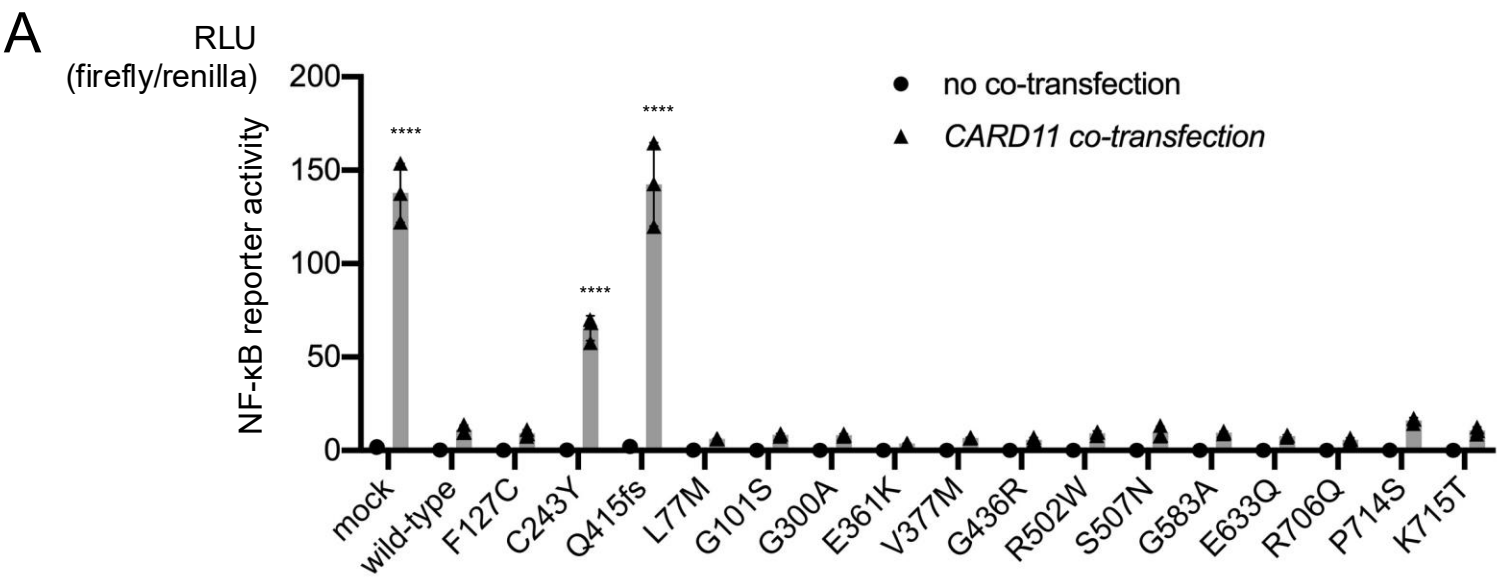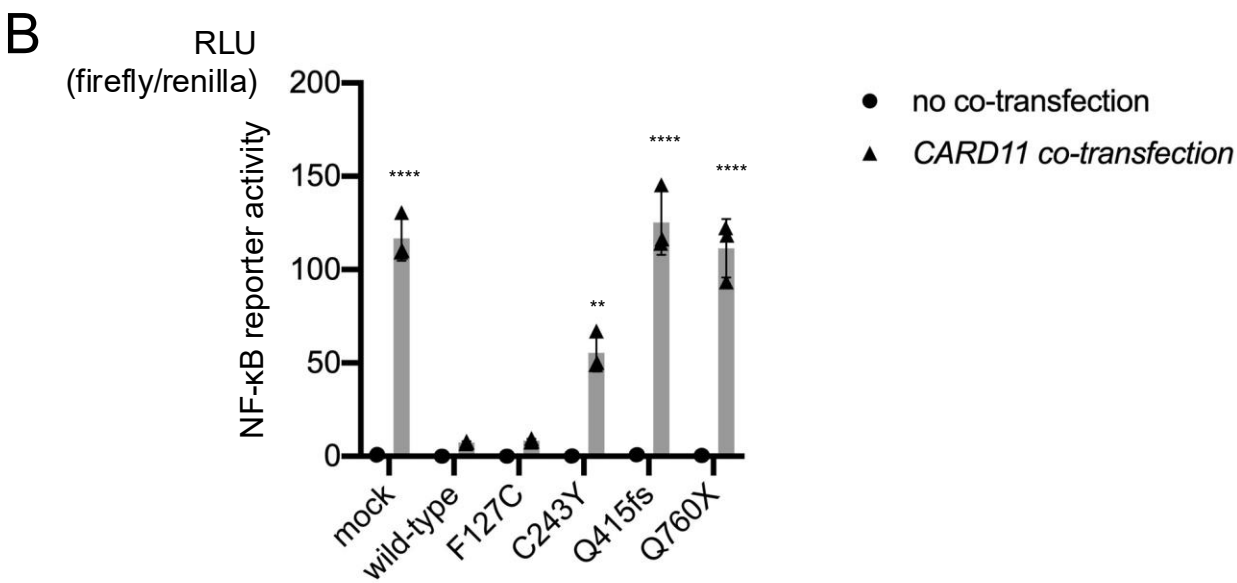

Supplement: Supplementary file 4 [file Image2.pdf]

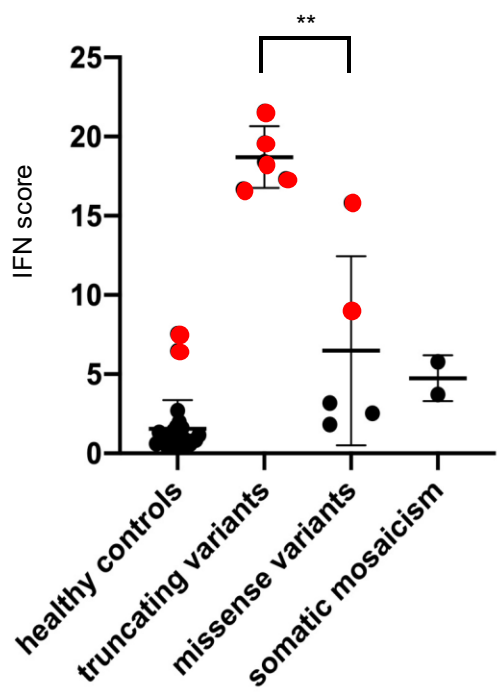

Supplement: Supplementary file 5 [file Image3.pdf]
