# Supplementary material for: Clinical characteristics and treatment strategies for A20 haploinsufficiency in Japan: a national epidemiological survey
Source: Front Immunol. 2025 Jun 12;16:1548042. doi: 10.3389/fimmu.2025.1548042 (PMC12197945; doi:10.3389/fimmu.2025.1548042)
Supplement: Supplementary file 6 [file Image4.pdf]

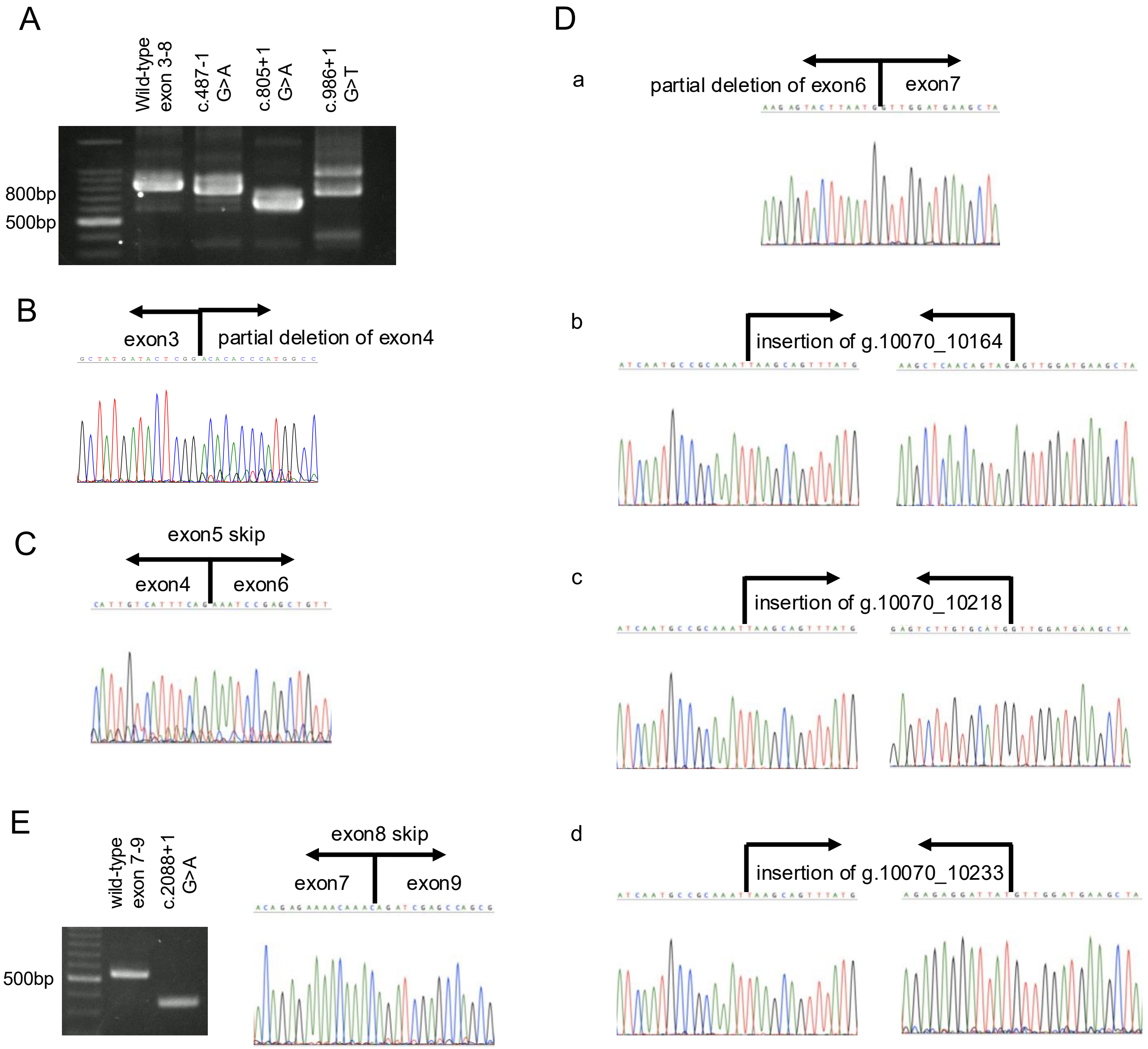
A
Wild-type exon 3-8
c.487-1 G>A
c.805+1 G>A
c.986+1 G>T
800bp
500bp
B
exon3
partial deletion of exon4
C
exon5 skip
exon4
exon6
D
a
partial deletion of exon6
exon7
b
insertion of g.10070_10164
c
insertion of g.10070_10218
d
insertion of g.10070_10233
E
wild-type exon 7-9
c.2088+1 G>A
500bp
exon8 skip
exon7
exon9
